# VHL suppresses UBE3B-mediated breast tumor growth and metastasis

**DOI:** 10.1038/s41419-024-06844-x

**Published:** 2024-06-24

**Authors:** Shuo Wang, Huiyan Li, Xiong Liu, Tingting Yin, Tingru Li, Miaomiao Zheng, Min Liu, Xiaoqian Meng, Jun Zhou, Yijie Wang, Yan Chen

**Affiliations:** 1 Shandong Provincial Key Laboratory of Animal Resistance Biology, Collaborative Innovation Center of Cell Biology in Universities of Shandong, Center for Cell Structure and Function, Institute of Biomedical Science, College of Life Sciences, Shandong Normal University, Jinan, Shandong 250014 China; 2 School of Medicine, Jinan University, Guangzhou, Guangdong 510632 China

**Keywords:** Breast cancer, Ubiquitylation

## Abstract

Protein homeostasis is predominantly governed through post-translational modification (PTM). UBE3B, identified as an oncoprotein, exhibits elevated protein levels in breast cancer. However, the impact of PTM on UBE3B remains unexplored. In this study, we show that VHL is a bona fide E3 ligase for UBE3B. Mechanistically, VHL directly binds to UBE3B, facilitating its lysine 48 (K48)-linked polyubiquitination at K286 and K427 in a prolyl hydroxylase (PHD)-independent manner. Consequently, this promotes the proteasomal degradation of UBE3B. The K286/427R mutation of UBE3B dramatically abolishes the inhibitory effect of VHL on breast tumor growth and lung metastasis. Additionally, the protein levels of UBE3B and VHL exhibit a negative correlation in breast cancer tissues. These findings delineate an important layer of UBE3B regulation by VHL.

## Introduction

It has been well-documented that dysfunctions of the E3 ligase UBE3B usually lead to Kaufman Oculocerebrofacial Syndrome (KOS), an autosomal recessive developmental disorder characterized by hypotonia, developmental delay, microcephaly, intellectual retardation, distinctive facial dysmorphic features, and low cholesterol levels [1–3]. Accumulating evidence suggests that UBE3B plays a crucial role in cancer progression and drug resistance [4–6]. TRIB3, through interaction with MYC, suppresses UBE3B-mediated MYC ubiquitination and degradation. This interaction results in MYC accumulation, subsequently promoting the proliferation and self-renewal of lymphoma cells [4]. Conversely, UBE3B ablation dramatically diminishes the proliferative capacity, colony formation, and resistance to the clinical alkylator temozolomide (TMZ) [5, 6]. Moreover, our recent findings indicate that UBE3B promotes breast cancer growth and metastasis by counteracting VHL-mediated HIF-2α degradation [7]. These pieces of evidence suggest that UBE3B functions as an oncoprotein or tumor suppressor in a context-dependent manner. Notably, UBE3B protein levels are increased in breast tumor tissues, as evidenced by our previous data [7] and immunohistochemistry data from HPA (Human Protein Atlas, https://www.proteinatlas.org/) [8]. However, the mechanism underlying the upregulation of UBE3B protein levels in breast cancer remains unexplored.

Protein homeostasis is primarily regulated by post-translational modification (PTM), among which ubiquitination plays a dominant role [9–11]. The E3 ligase Von Hippel-Lindau (VHL) is an important tumor suppressor that is dysfunctional in multiple cancers, including breast cancer [12, 13]. Breast cancer cells display relatively lower VHL protein levels as compared to normal breast cells [14, 15], although mutations of VHL are rare in breast cancer [16]. Hypoxia-inducible factor 2α (HIF-2α) is the best-characterized protein clearly linked to VHL-associated tumors [12, 17, 18]. However, recently developed HIF-2α inhibitors could only suppress the growth of certain renal cell carcinomas but not others [9, 19, 20], suggesting the existence of unidentified VHL substrates. Therefore, identifying novel VHL substrates could benefit the clinical outcome of breast cancer patients.

In the present study, we demonstrate that VHL regulates UBE3B turnover. VHL interacts with UBE3B, promoting its polyubiquitination at lysine (K) 286 and K427, leading to subsequent proteasomal degradation. VHL-mediated UBE3B degradation suppresses breast tumor growth and metastasis. Importantly, the protein levels of UBE3B and VHL exhibit a negative correlation in breast cancer tissues. Our study uncovers the functional role and regulatory mechanism of UBE3B in breast cancer progression, providing a potential diagnostic biomarker and therapeutic target for breast cancer.

## Results

### UBE3B directly interacts with VHL

**Fig. 1:**
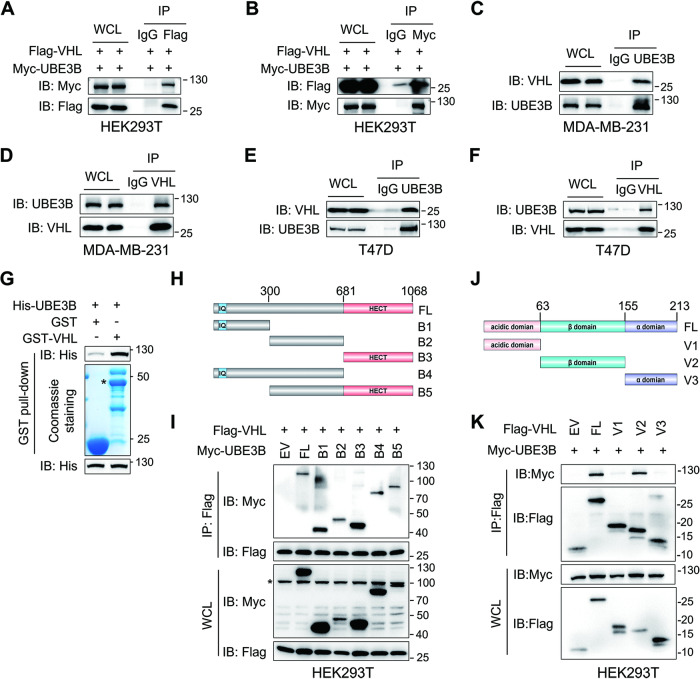
VHL directly interacts with UBE3B. **A**, **B** HEK293T cells transfected with vectors expressing Flag-VHL and Myc-UBE3B were subjected to Co-IP assays using the anti-Flag (**A**) and anti-Myc (**B**) antibodies. **C**, **D** Co-IP of endogenous VHL with UBE3B (**C**) and vice versa (**D**) in MDA-MB-231 cells treated with 10 μM MG132 for 6 h before harvest. **E**, **F** Co-IP of endogenous VHL with UBE3B (**E**) and vice versa (**F**) in T47D cells treated with 10 μM MG132 for 6 h before harvest. **G** Pull-down assay was performed using purified His-UBE3B and GST-VHL. **H**, **I** Mapping UBE3B regions binding to VHL by Co-IP assays. **J**, **K** Mapping VHL regions binding to UBE3B by co-IP assays. Asterisk (*) indicates the non-specific band. WCL, whole-cell lysate.

To elucidate the mechanism by which UBE3B protein levels are upregulated in breast cancer, we scrutinized the published liquid chromatography-tandem mass spectrometry (LC-MS/MS) data obtained from MDA-MB-231 cells stably expressing Flag-UBE3B (Table S1) [7]. This analysis identified the E3 ligase VHL as a potential UBE3B-interacting protein (Fig. S1A; Table S1). To validate the physical interaction between VHL and UBE3B, coimmunoprecipitation (Co-IP) assays were conducted. Reciprocal interaction between ectopically expressed Myc-UBE3B and Flag-VHL was observed in both HEK293T cells and HeLa cells (Fig. 1A, B; Fig. S1B). This interaction was further confirmed at the endogenous level in two human breast cancer cell lines-metastatic MDA-MB-231 cells (Fig. 1C, D) and non-metastatic T47D cells (Fig. 1E, F). To examine whether VHL directly interacts with UBE3B, we performed GST pull-down assays using purified proteins. The results indicated that GST-VHL, but not GST alone, interacted with His-UBE3B (Fig. 1G). VHL typically interacts with hydroxylated proteins, which are catalyzed by prolyl hydroxylases (EGLN1, EGLN2, and EGLN3) [9, 18, 21, 22], encouraging us to investigate UBE3B hydroxylation. Surprisingly, UBE3B was not hydroxylated and had no interaction with EGLN1, EGLN2, or EGLN3 (Fig. S1C, D). Taken together, these findings indicate that VHL interacts with UBE3B in a hydroxylation-independent manner.

Next, we aimed to identify the specific domains facilitating the UBE3B-VHL interaction. Co-IP assays with full-length (FL) or truncated Myc-UBE3B unexpectedly revealed that all truncated mutants retained the ability to bind VHL (Fig. 1H, I). Given that VHL comprises a β-domain flanked by an N-terminal acidic domain and an α-domain at the C-terminus [23] and that β-domain of VHL is critical for substrate binding [23, 24], truncated mutants expressing Flag-tagged acidic domain, β-domain, and α-domain were generated, respectively (Fig. 1J). Full-length Flag-VHL and these mutants were transiently transfected into HEK293T cells, followed by Co-IP assays. These assays demonstrated that both full-length VHL and the β-domain of VHL associated with UBE3B, whereas the acidic domain and α-domain of VHL failed to interact with UBE3B (Fig. 1K). Thus, the β-domain of VHL is required for the VHL-UBE3B interaction. Collectively, these findings demonstrate that VHL directly binds to UBE3B via its β-domain.

### VHL decreases UBE3B protein stability in breast cancer cells

VHL is a well-known E3 ubiquitin ligase that has been reported to degrade several substrates, including HIF-α [9, 18, 22], ZHX2 (Zinc Fingers and Homeoboxes 2) [25], KLF4 (Krüppel-like factor 4) [26], and SFMBT1 (Scm Like With Four Mbt Domains 1) [27]. UBE3B, involved in various types of ubiquitination on substrates, exhibits a positive or negative effect on protein stability [4, 7, 28, 29]. Given the direct interaction between VHL and UBE3B, we aimed to determine the reciprocal influence between VHL and UBE3B in HEK293T cells. Overexpression of Flag-VHL downregulated Myc-UBE3B protein levels, while the protein levels of Flag-VHL remained unaffected by ectopically expressed Myc-UBE3B (Fig. S2A). Thus, VHL is a potential E3 ligase for UBE3B.

**Fig. 2:**
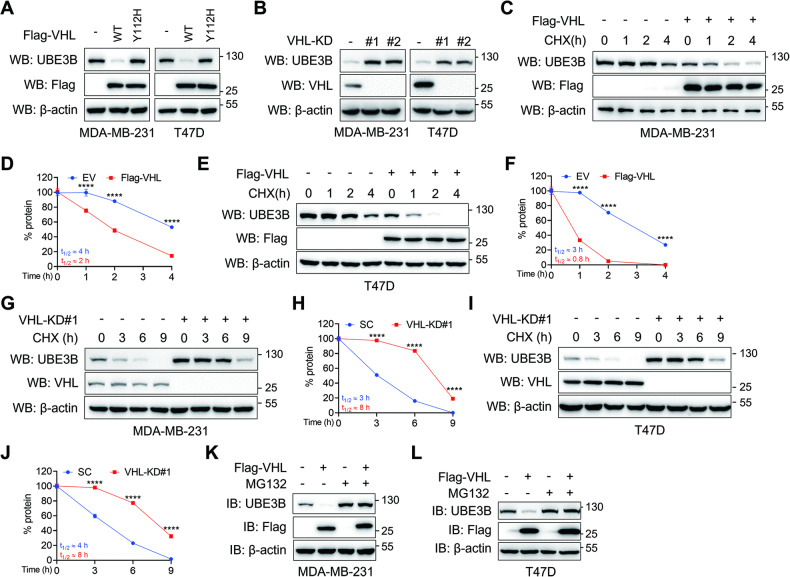
VHL decreases UBE3B protein levels in breast cancer cells. **A** Protein levels of endogenous UBE3B were assessed in MDA-MB-231 and T47D cells stably expressing either wild-type (WT) or catalytically inactive (Y112H) Flag-VHL. **B** Protein levels of endogenous UBE3B in control or VHL-knockdown (VHL-KD) MDA-MB-231 and T47D cells. **C**–**F** The half-life of UBE3B protein in MDA-MB-231 (**C**, **D**) or T47D (**E**, **F**) cells expressing Flag-VHL. Cells were treated with 10 μg/mL CHX for the indicated time (mean ± SD, *n* = 3). ****P < 0.0001, by 2-way ANOVA Tukey’s multiple comparisons test. **G**–**J** The half-life of UBE3B protein was analyzed in scrambled shRNA (SC) or VHL-KD MDA-MB-231 (G, H) or T47D (**I**, **J**) cells. Cells were treated with 10 μg/mL CHX for the indicated time (mean ± SD, *n* = 3). ****P < 0.0001, by 2-way ANOVA Tukey’s multiple comparisons test. **K**, **L** UBE3B protein levels in Flag-VHL-expressing MDA-MB-231 (**K**) and T47D (**L**) cells with or without MG132 treatment. EV, empty vector.

To substantiate the impact of VHL on UBE3B protein stability, wild-type (WT) and catalytically inactive (Y112H) Flag-VHL was ectopically expressed in MDA-MB-231 and T47D breast cancer cells [30]. The overexpression of WT, but not Y112H, Flag-VHL led to a dramatic downregulation of UBE3B protein levels in these cells, suggesting that VHL modulates UBE3B protein levels in an enzymatic manner (Fig. 2A). Next, we depleted VHL via two independent VHL shRNAs (shVHL#1 and shVHL#2) in MDA-MB-231 and T47D cells and found that VHL ablation in these cells markedly upregulated endogenous UBE3B protein levels (Fig. 2B). VHL directly binds the HNF-4α promoter to modulate its transcription, prompting us to evaluate the effect of VHL on UBE3B transcription [31]. RT-qPCR results indicated that VHL depletion had no impact on UBE3B mRNA levels (Fig. S2B, C). Together, these results suggest that VHL may regulate UBE3B protein stability in breast cancer cells.

To investigate whether VHL modulates UBE3B stability, we conducted cycloheximide (CHX) chasing assays. MDA-MB-231 and T47D cells transfected with empty vector (EV) or Flag-VHL were treated with CHX to determine the protein half-life of UBE3B. Compared to the control, cells stably expressing Myc-VHL displayed a reduced half-life of UBE3B (Fig. 2C–F). Conversely, VHL knockdown in MDA-MB-231 and T47D cells greatly increased the half-life of UBE3B (Fig. 2G–J). Furthermore, VHL-mediated UBE3B downregulation in breast cancer cells could be restored by the proteasome inhibitor MG132 (Fig. 2K, L), indicating that VHL modulates UBE3B protein levels through the proteasomal system. Notably, the HIF hydroxylase inhibitor dimethyloxallyl glycine (DMOG) did not impede VHL-mediated UBE3B downregulation, supporting the conclusion that VHL binds and modulates UBE3B in a hydroxylase-independent manner (Fig. S2D). Taken together, VHL regulates UBE3B protein stability in breast cancer cells.

### VHL inhibits UBE3B-mediated breast cancer cell proliferation and invasion

**Fig. 3:**
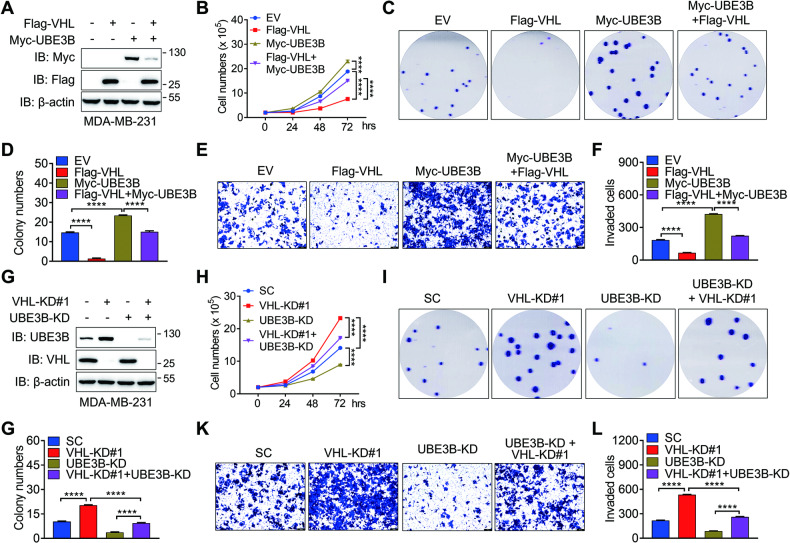
VHL inhibits the UBE3B-mediated tumorigenic potential of breast cancer cells. **A** Protein levels of Flag-VHL and Myc-UBE3B in the stable MDA-MB-231 cell line expressing Flag-VHL or Myc-UBE3B. **B** The cell proliferation rate was measured in the indicated MDA-MB-231 cells (mean ± SD, *n* = 3). ****P < 0.0001, by 2-way ANOVA Tukey’s multiple comparisons test. hrs, hours. **C**, **D** Colony formation was conducted in the indicated MDA-MB-231 cells, and representative images from three experiments were shown in (**C**). Colony numbers (**D**) were quantified (mean ± SD, *n* = 3). ****P < 0.0001, by 1-way ANOVA Tukey’s multiple comparisons test. **E**, **F** Invasion assays were carried out in the indicated MDA-MB-231 cells, and representative images from three experiments were shown in (**E**). Invaded cell numbers were quantified in (**F**) (mean ± SD, *n* = 3). ****P < 0.0001, by 1-way ANOVA Tukey’s multiple comparisons test. **G** Protein levels of VHL and UBE3B were detected in control, VHL-KD#1, UBE3B-KD, and VHL- and UBE3B-double knockdown (VHL-KD#1 + UBE3B-KD) MDA-MB-231 cells. **H** Proliferation rate of control, V**H**L-KD#1, UBE3B-KD, and VHL-KD#1+UBE3B-KD MDA-MB-231 cells (mean ± SD, *n* = 3). ****P < 0.0001, by 2-way ANOVA Tukey’s multiple comparisons test. hrs, hours. **I**–**L** Colony formation (**I**, **J**) and invasion (**K**, **L**) assays were performed in control, VHL-KD#1, UBE3B-KD, and VHL-KD#1+UBE3B-KD MDA-MB-231 cells, and the representative images from three experiments were shown. Colony numbers (**J**) and invaded cell numbers (**L**) were quantified (mean ± SD, *n* = 3). ****P < 0.0001, by 1-way ANOVA Tukey’s multiple comparisons test. Scale bars: 100 μm. EV, empty vector.

Given the established role of UBE3B as an oncoprotein in breast cancer [7] and its regulation by VHL, we sought to examine whether VHL could inhibit UBE3B-mediated breast cancer cell proliferation, survival, and invasion. To this end, Flag-VHL and Myc-UBE3B were expressed in MDA-MB-231 and T47D cells alone or simultaneously (referred to as Flag-VHL + Myc-UBE3B) by generating stable cell lines with lentivirus (Fig. 3A; Fig. S3A). In line with previous report [7], UBE3B overexpression increased the ability of MDA-MB-231 and T47D cells to proliferate and form colonies (Fig. 3B–D; Fig. S3B–D). Importantly, this effect was partially but significantly attenuated by the presence of Flag-VHL (Fig. 3B–D; Fig. S3B–D). Additionally, Boyden chamber assays conducted in metastatic MDA-MB-231 cells demonstrated that UBE3B overexpression enhanced the invasive capacity of these cells, and this phenotype was markedly mitigated, at least partially, by VHL overexpression. (Fig. 3E, F). To further illustrate that VHL hinders the oncogenic potential of breast cancer cells through its regulation of UBE3B, we depleted UBE3B in MDA-MB-231 and T47D cells following VHL ablation (referred to as VHL-KD#1+UBE3B-KD) (Fig. 3G; Fig. S3E). The proliferation assays and colony growth assays showed that the enhanced oncogenic potential resulting from VHL knockdown could be effectively counteracted by UBE3B depletion (Fig. 3H–J; Fig. S3F–H). Consistently, VHL-KD#1+UBE3B-KD remarkably inhibited the increased invasive capacity of MDA-MB-231 cells caused by VHL depletion alone (Fig. 3K, L). Collectively, VHL suppresses breast cancer cell proliferation, survival, and invasion, at least partially, by inhibiting UBE3B.

### VHL inhibits UBE3B-mediated breast tumor growth and metastasis in mice

**Fig. 4:**
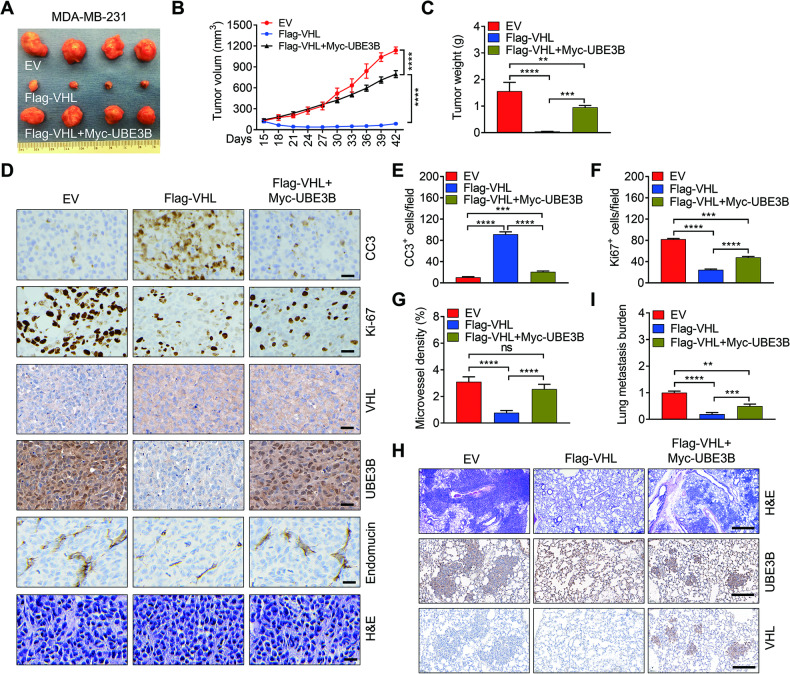
UBE3B alleviates VHL’s inhibitory effect on breast tumor growth and metastasis. **A**–**C** Tumor images (**A**), tumor growth curves (**B**), and tumor weight (**C**) of mice with orthotopic implantation of EV (empty vector), Flag-VHL, and Flag-VHL + Myc-UBE3B MDA-MB-231 cells into the mammary fat pad (mean ± SD, *n* = 4). ****P < 0.0001, by 2-way ANOVA Tukey’s multiple comparisons test (**B**) or 1-way ANOVA Tukey’s multiple comparisons test (**C**). **D**–**G** Representative H&E staining and immunohistochemical staining of Ki-67, CC3 (cleaved caspase-3), and endomucin in primary tumors (**D**). Scale bars: 100 μm. Ki-67-positive cell numbers (**E**), CC3-positive cell numbers (**F**), and microvessel density (**G**) were quantified. (mean ± SD, *n* = 4). ***P < 0.001, ****P < 0.0001, by 1-way ANOVA Dunnett’s multiple comparisons test. **H**, **I** Representative images of metastases and protein levels of UBE3B and VHL in the lungs analyzed by H&E staining and IHC staining (**H**), respectively. Lung metastases were quantified by qPCR (**H**) (mean ± SD, *n* = 4). **P < 0.01, ***P < 0.001, ****P < 0.0001, by 1-way ANOVA Tukey’s multiple comparisons test. Scale bars: 500 μm.

To evaluate the impact of VHL on UBE3B-mediated breast tumor growth and metastasis in vivo, control, Flag-VHL, and Flag-VHL + Myc-UBE3B MDA-MB-231 cells were implanted into the mammary fat pad of female nonobese diabetic-severe combined immunodeficiency (NOD-SCID) mice, respectively. As anticipated, Flag-VHL led to decreased spontaneous breast tumor growth, a phenomenon effectively counteracted by Myc-UBE3B (Fig. 4A–C). Immunohistochemistry (IHC) staining indicated that Flag-VHL markedly increased the number of cleaved caspase-3 (CC3)-positive cells in MDA-MB-231 tumors. This increase was rescued by Myc-UBE3B (Fig. 4D, E), suggesting that VHL promotes cell death by inhibiting UBE3B. Conversely, the number of Ki-67-positive cells was markedly downregulated by Flag-VHL, and this downregulation was restored by Myc-UBE3B (Fig. 4D, F), suggesting that VHL impedes cell proliferation by suppressing UBE3B. Similarly, control, VHL-KD#1, and VHL-KD#1+UBE3B-KD T47D cells were injected into the fat pad of female NOD-SCID mice. T47D tumor growth was significantly increased when VHL was depleted, and this augmented tumor growth was effectively attenuated by UBE3B ablation (Fig. S4A–C). In line with findings in MDA-MB-231 tumors, the increased cell proliferation and decreased cell death in VHL-KD#1 T47D tumors were alleviated by UBE3B depletion (Fig. S4D–F). Approximately 90% of breast cancer-related deaths are attributed to metastasis and the lung is one of the most common metastatic sites for breast cancer [32, 33], and UBE3B regulates *VEGFA* expression and breast cancer lung metastasis [7], we therefore investigated whether VHL controls spontaneous breast cancer metastasis to the lungs via UBE3B. First, we examined the microvessel density in primary tumors by IHC assays and found that the decreased microvessel density by VHL overexpression was restored by overexpressed UBE3B in MDA-MB-231 tumors (Fig. 4D, G), and the increased microvessel density by VHL knockdown was partially but significantly abolished by UBE3B depletion (Fig. S4D, G), suggesting a critical role of VHL-UBE3B axis in breast tumor angiogenesis. Next, we determined the lung metastasis burden by H&E staining and RT-qPCR. Compared to control mice, lung metastases were barely detectable in mice bearing Flag-VHL tumor (Fig. 4H, I). Intriguingly, the attenuation of lung metastasis by Flag-VHL was robustly restored by Myc-UBE3B (Fig. 4H, I). The protein levels of Flag-VHL, Myc-UBE3B, VHL, and UBE3B in primary tumors were assessed by western blotting (Fig. S4H, I). Consistent with the western blotting results, the protein levels of UBE3B and VHL in primary tumors and metastatic lung tissues were negatively correlated, as evidenced by IHC staining (Fig. 4D, H; Fig. S4D). In summary, these findings indicate that VHL suppresses breast tumor growth and lung metastasis by inhibiting UBE3B.

### VHL ubiquitinates UBE3B at lysine residues 286 and 427

**Fig. 5:**
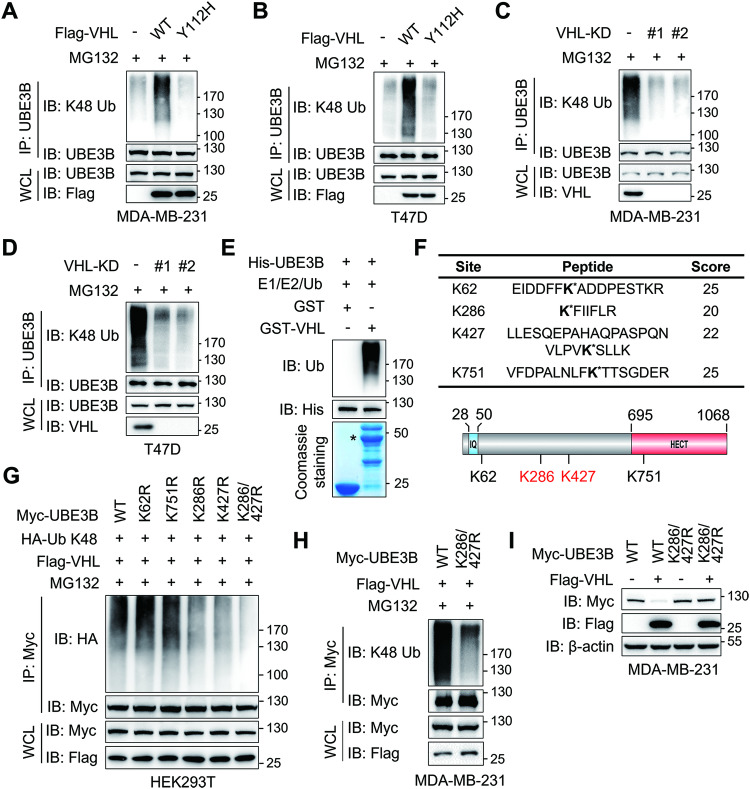
VHL ubiquitylates UBE3B at lysine residues 286 and 427. **A**, **B** Ubiquitination of endogenous UBE3B in MDA-MB-231 cells (**A**) and T47D cells (**B**) with ectopic expression of WT or Y112H Flag-VHL. **C**, **D** Ubiquitination of endogenous UBE3B in control or VHL knockdown MDA-MB-231 cells (**C**) and T47D cells (**D**). **E** In vitro ubiquitination of His-UBE3B by GST-VHL. **F** HEK293T cells were co-transfected with Myc-UBE3B, Flag-VHL, and HA-ubiquitin. Immunoprecipitated Myc-UBE3B was subjected to mass spectrometry analysis to recover potential ubiquitination sites. The identified ubiquitination sites were indicated with an asterisk. **G** Ubiquitination of immunoprecipitated WT or mutant Myc-UBE3B by Flag-VHL in HEK293T cells treated with 10 μM MG132 for 6 h before harvest. **H** Ubiquitination of immunoprecipitated WT or K286/427R mutant (DM) Myc-UBE3B by Flag-VHL in MDA-MB-231 cells treated with 10 μM MG132 for 6 h before harvest. **I** Protein levels of WT or DM Myc-UBE3B in MDA-MB-231 cells with or without Flag-VHL. WCL whole-cell lysate.

Since VHL controls UBE3B stability through the proteasomal system (Fig. 2K, L), we carried out in vivo ubiquitination assays to determine whether VHL governs UBE3B turnover through ubiquitination. Considering that VHL catalyzes both K48- and K63-linked polyubiquitination [9, 34], we first investigated the VHL-mediated linkage of ubiquitin chains on UBE3B. Our results indicated that VHL remarkably increased the K48-linked polyubiquitination of UBE3B (Fig. S5A). Further investigation in MDA-MB-231 and T47D cells expressing WT or Y112H Flag-VHL revealed a substantial increase in K48-linked polyubiquitination of UBE3B by WT but not Y112H Flag-VHL (Fig. 5A, B). Consistently, both VHL-KD#1 and VHL-KD#2 dramatically downregulated K48-linked polyubiquitination of UBE3B in MDA-MB-231 and T47D cells (Fig. 5C, D). Furthermore, in vitro ubiquitination assay demonstrated that purified His-UBE3B was ubiquitinated by purified GST-VHL (Fig. 5E). Taken together, these results confirm VHL as a bona fide UBE3B E3 ligase that conjugates K48-linked polyubiquitination to UBE3B.

To pinpoint the potential ubiquitination sites of UBE3B by VHL, immunoprecipitated Myc-UBE3B from HEK293T cells expressing Myc-UBE3B, Flag-VHL as well as HA-ubiquitin was subjected to liquid chromatography-tandem mass spectrometry (LC-MS/MS) analysis. LC-MS/MS results indicated that K62, K286, K427, and K751 of Myc-UBE3B appeared to be the major ubiquitination sites by VHL (Fig. 5F; Fig. S5B). To validate the role of these lysine residues in UBE3B ubiquitination, Myc-UBE3B vectors with single K-to-arginine (R) mutations were constructed. Both K286R and K427R mutations partially but significantly attenuated VHL-mediated UBE3B ubiquitination in HEK293T cells compared to WT Myc-UBE3B (Fig. 5G). Then, we generated K286 and K427 double mutant (K286R/K427R, referred to as DM) Myc-UBE3B and found that K286R/K427R almost completely diminished UBE3B ubiquitination by VHL (Fig. 5G). To further demonstrate that UBE3B is ubiquitinated by VHL at K286 and K427 in breast cancer cells, MDA-MB-231 cells were transfected with vectors expressing Flag-VHL along with WT or DM Myc-UBE3B. While the protein levels of WT and DM Myc-UBE3B were comparable in MDA-MB-231 cells treated with 10 μM MG132, K48-linked ubiquitination of WT but not DM Myc-UBE3B was markedly increased by Flag-VHL (Fig. 5H). Moreover, DM Myc-UBE3B conferred complete resistance to VHL-mediated degradation (Fig. 5I). Intriguingly, mutations of K286 and K427 have been identified in uterine papillary serous carcinoma, skin carcinoma, or tumors of unknown species (Fig. S5C, D), as evidenced by datasets from Integrative Onco Genomics (IntOGen, https://www.intogen.org/search) [35], Catalogue Of Somatic Mutations In Cancer (COSMIC, https://cancer.sanger.ac.uk/cosmic) [36], and cBio Cancer Genomics Portal (cBioPortal, https://www.cbioportal.org/) [37]. Collectively, these findings demonstrate that K286 and K427 are key sites for VHL-mediated UBE3B ubiquitination in breast cancer cells.

### VHL-mediated UBE3B ubiquitination is indispensable for VHL’s inhibitory effect in breast cancer cells

**Fig. 6:**
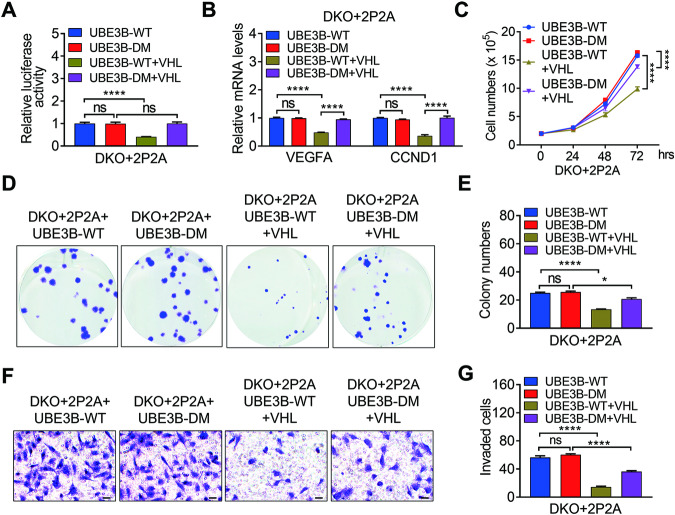
VHL-mediated UBE3B ubiquitination is required for VHL’s inhibitory effect in breast cancer cells. **A**, **B** Luciferase activity (**A**) and mRNA levels of VEGFA and CCND1 (**B**) were determined in DKO+2P2A + Myc-UBE3B-WT, DKO+2P2A + Myc-UBE3B-DM, DKO+2P2A + Myc-UBE3B-WT + Flag-VHL, and DKO+2P2A + Myc-UBE3B-DM + Flag-VHL MDA-MB-231 cells (mean ± SD, *n* = 3). ****P < 0.0001, by 1-way ANOVA Tukey’s multiple comparisons test. **C**–**F** Proliferation (**C**), colony formation (**D**, **E**) and invasion assays (**F**, **G**) were performed using DKO+2P2A + Myc-UBE3B-WT, DKO+2P2A + Myc-UBE3B-DM, DKO+2P2A + Myc-UBE3B-WT+Flag-VHL, and DKO+2P2A + Myc-UBE3B-DM + Flag-VHL MDA-MB-231 cells (mean ± SD, *n* = 3). ****P < 0.0001, by 2-way ANOVA Tukey’s multiple comparisons test (**C**) or 1-way ANOVA Tukey’s multiple comparisons test (**E**, **G**). ns, no significance. DKO, HIF-1α/HIF-2α double knockout. 2P2A, HIF-1α-P402/564A (HIF-1α-P2A) and HIF-2α-P405/531A (HIF-2α-P2A). DM, UBE3B-K286/427R.

VHL promotes the ubiquitination and degradation of UBE3B, which stabilizes the oncoprotein HIF-2α in breast cancer [7]. This finding led us to determine whether VHL-mediated UBE3B ubiquitination affects the oncogenic potential of breast cancer cells. To this end, we first examined HIF-2α transcriptional activity using an HIF luciferase reporter containing 2 x HREs from the human *VEGFA* (*Vascular Endothelial Growth Factor A*) gene. This reporter has been reported to be regulated by both HIF-1α and HIF-2α [7, 33]. We utilized HIF-1α and HIF-2α double knockout (DKO) MDA-MB-231 cells or T47D cells expressing proline (P) hydroxylation-resistant HIF-1α P402A/P564A (referred to as HIF-1α-P2A) and HIF-2α P405A/P531A (referred to as HIF-2α-P2A) [38, 39] to eliminate the influence of endogenous HIF-1α and HIF-2α since HIF-1α and HIF-2α are degraded by VHL [9, 18, 22]. The protein levels of HIF-1α-P2A and HIF-2α-P2A were comparable to those of endogenous HIF-1α and HIF-2α, respectively (Fig. S6A, B). Hereafter, DKO+HIF-1α-P2A+HIF-2α-P2A cells are referred to as DKO+2P2A cells. Both WT and DM Myc-UBE3B significantly stimulated HIF luciferase reporter activity in DKO+2P2A MDA-MB-231 cells and DKO+2P2A T47D cells (Fig. 6A; Fig. S6C). However, WT, but not DM Myc-UBE3B-stimulated HIF luciferase reporter activity was remarkably inhibited by Flag-VHL in these cells (Fig. 6A; Fig. S6C). Consistently, Flag-VHL overexpression resulted in decreased WT Myc-UBE3B and HIF-2α-P2A (Fig. S6A, B). Moreover, VHL downregulated WT but not DM UBE3B-mediated transcription of HIF-2α target genes *VEGFA* and *CCND1* (*Cyclin D1*), which are critical for angiogenesis and cell proliferation, respectively [7] (Fig. 6A; Fig. S6D). These findings supported the conclusion that the VHL-UBE3B axis controls cell proliferation, microvessel density, and lung metastasis of breast cancer (Fig. 4D–I; Fig. S4D–I). Together, VHL modulates HIF-2α stability by ubiquitinating and degrading UBE3B.

Next, we assessed the effect of VHL on the UBE3B-HIF-2α axis-mediated breast cancer cell proliferation, survival, and invasion. The proliferation of DKO+2P2A+UBE3B-WT MDA-MB-231 cells or T47D cells was markedly inhibited by VHL (Fig. 6C; Fig. S6E). In contrast, VHL only moderately affected the proliferation of DKO+2P2A+UBE3B-DM MDA-MB-231 cells or T47D cells (Fig. 6C; Fig. S6E), suggesting that VHL has substrates other than HIF-1α, HIF-2α and UBE3B. Consistent with these findings, colony-formation assays and Boyden chamber assays also demonstrated that VHL remarkably ameliorated UBE3B-WT-mediated survival and invasion of MDA-MB-231 cells or T47D cells, whereas it had a weak effect on DKO+2P2A+UBE3B-DM MDA-MB-231 cells or T47D cells (Fig. 6D–G; Fig. S6F, G). Taken together, VHL promotes UBE3B and subsequent HIF-2α degradation and consequently inhibits the oncogenic potential of breast cancer cells.

### VHL-mediated UBE3B ubiquitination is critical for VHL in suppressing breast tumor growth and metastasis

**Fig. 7:**
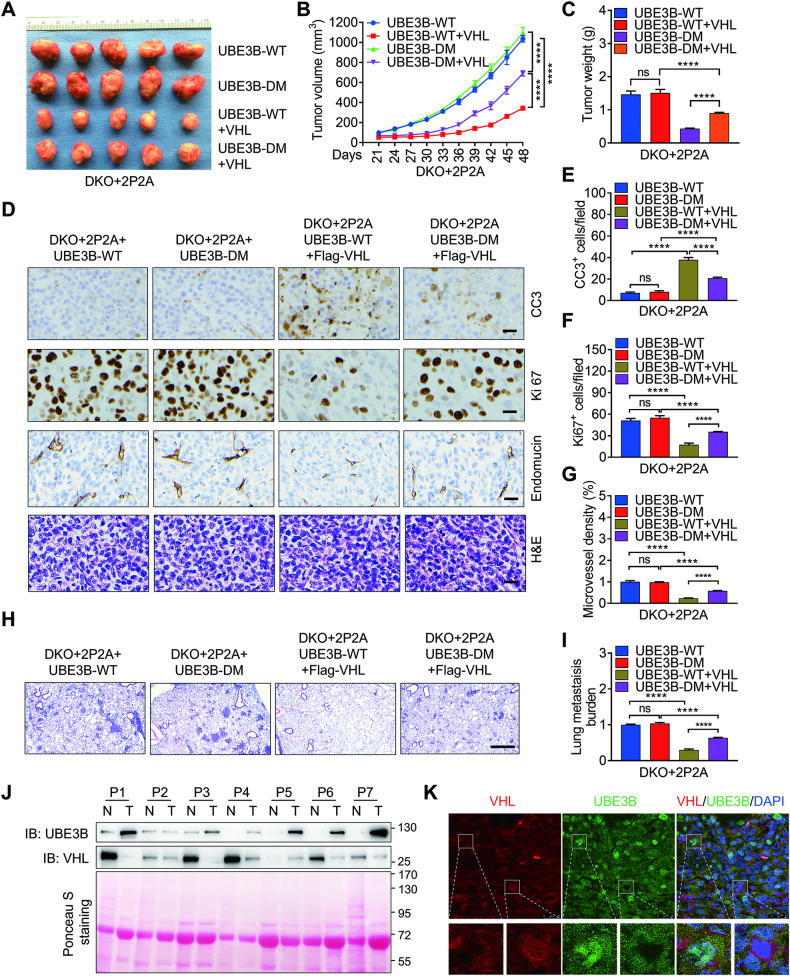
VHL-mediated UBE3B ubiquitination is essential for VHL in suppressing breast tumor growth and metastasis. **A**–**C** Tumor images (**A**), tumor growth curves (**B**), and tumor weight (**C**) of mice bearing DKO+2P2A + Myc-UBE3B-WT, DKO+2P2A + Myc-UBE3B-DM, DKO+2P2A + Myc-UBE3B-WT+Flag-VHL, and DKO+2P2A + Myc-UBE3B-DM+Flag-VHL T47D tumors (mean ± SD, *n* = 5). ****P < 0.0001, by 2-way ANOVA Tukey’s multiple comparisons test (**B**) or 1-way ANOVA Tukey’s multiple comparisons test (**C**). **D**–**G** Representative H&E and immunohistochemical staining of Ki-67, CC3, and endomucin in primary tumors. Scale bars: 100 μm. Ki-67-positive cell numbers (**E**), CC3-positive cell numbers (**F**), and microvessel density (**G**) were quantified. (mean ± SD, *n* = 5). ***P < 0.001, ****P < 0.0001, by 1-way ANOVA Dunnett’s multiple comparisons test. ns no significance. **H**, **I** DKO+2P2A + Myc-UBE3B-WT, DKO+2P2A + Myc-UBE3B-DM, DKO+2P2A + Myc-UBE3B-WT+Flag-VHL, and DKO+2P2A + Myc-UBE3B-DM+Flag-VHL MDA-MB-231 cells were injected into mice through tail vein. Representative H&E-stained lung sections (**H**) and quantification of lung metastases by qPCR (**I**) (mean ± SD, *n* = 4). ****P < 0.0001, 1-way ANOVA Tukey’s multiple comparisons test. Scale bars: 500 μm. **J** Immunoblots of lysates from paired normal (N) and breast cancer tissues (T). **K** Immunostaining of UBE3B and VHL in human breast cancer tissues. Scale bars: 25 μm.

Our next objective was to investigate VHL’s role in UBE3B-mediated breast tumor growth and metastasis in vivo. To achieve this, we injected the T47D stable cell lines described in Fig. S6B–F into the mammary fat pads of female NOD-SCID mice. As expected, the tumor volume and weight were comparable between DKO+2P2A+UBE3B-WT and DKO+2P2A+UBE3B-DM T47D tumors (Fig. 7A–C). Importantly, the overexpression of Flag-VHL significantly suppressed the growth of DKO+2P2A+UBE3B-WT T47D tumors (Fig. 7A–C), while the growth of DKO+2P2A+UBE3B-DM T47D tumors was moderately inhibited by Flag-VHL (Fig. 7A–C). Furthermore, Flag-VHL significantly upregulated CC3-positive cell numbers and microvessel density but downregulated Ki-67-positive cell numbers in DKO+2P2A+UBE3B-WT T47D (Fig. 7D–G). However, Ki-67-positive cell numbers, microvessel density, and CC3-positive cell numbers in DKO+2P2A+UBE3B-DM T47D tumors were minimally affected by Flag-VHL (Fig. 7D–G). The protein levels of Myc-UBE3B and Flag-VHL in T47D tumors were detected by western blotting (Fig. S7A). To demonstrate the effect of VHL on UBE3B-mediated lung metastasis of breast cancer cells, metastatic MDA-MB-231 cells described in Fig. 6A–F were injected into female NOD-SCID mice via the tail vein to rule out the possibility that reduced lung metastasis is due to small primary tumors. Flag-VHL overexpression dramatically inhibited angiogenesis and the lung metastases of DKO+2P2A+UBE3B-WT MDA-MB-231 tumors, but not DKO+2P2A+UBE3B-DM MDA-MB-231 tumors (Fig. 7H, I). Collectively, these results indicate that VHL inhibits UBE3B-mediated breast tumor growth and lung metastasis in mice. To explore the potential relevance of VHL and UBE3B in human breast cancer, we extracted cell lysates from breast tumors and adjacent normal breast tissues for western blotting. We observed decreased VHL protein levels in tumors, coinciding with upregulated UBE3B protein levels (Fig. 7J), suggesting a negative correlation between the protein levels of VHL and UBE3B in breast cancer. To further precisely outline the expression patterns of UBE3B and VHL in breast cancer cells, we performed immunostaining in breast cancer tissues. As expected, the results showed that about 45% of breast cancer cells displayed a negative correlation between the protein levels of UBE3B and VHL (Fig. 7K; Fig. S7B). Taken together, these data convincingly demonstrate that VHL inhibits breast tumor growth and metastasis by promoting UBE3B degradation (Fig. S7C).

## Discussion

In the present study, we identified UBE3B as a bona fide VHL substrate in breast tumors. Mechanistically, VHL directly interacts with UBE3B via its β domain, promoting the ubiquitination of UBE3B at lysine residues 286 and 427 and subsequent degradation. Consequently, UBE3B-induced HIF-2α stability and transcriptional activity were alleviated. In addition, we showed that UBE3B overexpression robustly attenuates VHL’s inhibitory effects on breast cancer cell proliferation, colony formation, and invasion in vitro, and breast tumor growth and lung metastasis in mice. Importantly, the protein levels of VHL and UBE3B have a negative correlation in breast tumors. Therefore, UBE3B emerges as a potential therapeutic target in breast cancer.

Previous studies have demonstrated that UBE3B depletion significantly suppresses glioblastoma cell proliferation and survival and sensitizes these cells to the anticancer drug TMZ [5, 6]. Recently, we demonstrated that UBE3B promotes breast tumor growth and metastasis by stabilizing HIF-2α stability without affecting its mRNA levels [7]. These findings indicate that UBE3B may function as an oncoprotein in cancer. However, the regulatory mechanism governing UBE3B itself in cancer remains unexplored. Our current findings uncovered that VHL is a bona fide E3 ligase for UBE3B in breast cancer. It is important to emphasize how UBE3B determines the types of ubiquitin chain covalently conjugated to its substrates, given its catalysis of both K63- and K48- linked polyubiquitination on HIF-2α and MYC, respectively [4, 7]. One possibility is the auxiliary factors or post-translationally modifications of UBE3B influence its preference for ubiquitin chain types [40]. Thus, it is critical to identify novel UBE3B partners. Degrons are short linear motifs bound by E3 ligases to target substrates for degradation [41, 42]. In contrast to MYC and HIF-2α, UBE3B has no impact on the VHL ubiquitination and stability, suggesting that UBE3B substrates may possess specific degrons for UBE3B-mediated ubiquitination. Further investigations are needed to determine whether UBE3B substrates contain degrons.

VHL, an important tumor suppressor, exhibits ubiquitous expression across various epithelial cell types, including renal tubule epithelial cells and breast epithelium [43]. VHL is usually mutant in a variety of cancers, such as clear cell carcinoma of renal cancer (ccRCC) [12, 15]. While VHL mutations are rare in breast cancer, their protein levels are lower than those in normal breast cells [14–16]. The canonical substrates of VHL are HIF-1α and HIF-2α [44]. However, growing evidence indicates that VHL has substrates other than HIF-1α and HIF-2α [12]. In this study, UBE3B is unveiled as a novel substrate of VHL, providing additional therapeutic options for cancer treatment. Hydroxylation of proline residues by prolyl hydroxylases (PHDs) is pivotal for VHL to recognize its substrates, such as HIF-α, ZHX2, and TBK1 (TANK-binding kinase 1) [25, 45]. However, VHL recognizes and ubiquitinates UBE3B in a PHD-independent manner. This strengthens the understanding that VHL can ubiquitinate proteins independently of PHDs and oxygen, as seen with AURKA (Aurora A kinase) and HDAC6 (Histone Deacetylase 6) [46, 47]. HIF-1α mainly responds to acute or transient hypoxia, while HIF-2α plays a dominant role in chronic hypoxia [48]. UBE3B specifically stabilizes HIF-2α but not HIF-1α, highlighting its critical role in chronic hypoxia in solid tumors.

The Hypoxia-PHDs-VHL axis plays a pivotal role in HIF-2α stability, but hypoxia alone is insufficient to confer HIF-2α resistance to proteasomal degradation. Several proteins, such as Int6 (Integration site 6, also known as eIF3e or p48), USP37 (Ubiquitin Specific Peptidase 37), and Sirt7 (Sirtuin 7), influence HIF-2α stability in hypoxia-and VHL-independent manners [49–51]. These findings suggest that, in addition to VHL, other E3 ubiquitin ligases must exist to modulate HIF-2α in a hypoxia-independent manner, although they still have not been identified. Additionally, UBE3B also stabilizes HIF-2α even with mutations at proline residues 405 and 531(Fig. S6A, B), which are typically hydroxylated by PHDs and recognized by VHL, suggesting that UBE3B can stabilize HIF-2α by antagonizing VHL and other unknown E3 ligases [7]. Identifying the potential E3 ligase (s) promoting HIF-2α degradation, whose activity is antagonized by UBE3B, is crucial to deciphering UBE3B’s functions in cancer. It is still challenging to design small molecules for transcription factors, including HIF-2α, due to their structural disorder and the lack of binding pockets [52]. Recently developed HIF-2α inhibitors PT2399 and PT2385 only have efficacy in specific renal cell carcinomas, but not in other types of cancer [19, 20, 53, 54]. Since UBE3B can effectively block VHL- and other E3 ligases-mediated HIF-2α degradation, it may provide a promising alternative strategy for targeting HIF-2α. Proteolysis-targeting chimeras (PROTACs) have emerged to be a novel therapeutic method [55, 56]. Therefore, the development of VHL-based PROTACs targeting UBE3B may offer potential benefits for breast cancer patients.

In summary, VHL promotes UBE3B degradation under both hypoxic and normoxic conditions, but HIF-2α is degraded by VHL only in normoxia (Fig. S7C). UBE3B, operating in an oxygen-independent manner, antagonizes VHL and other E3 ligases-mediated HIF-2α degradation (Fig. S7C). These findings unveil a complex regulatory network involving VHL, UBE3B, and HIF-2α, offering valuable insights into potential therapeutic interventions for breast cancer.

## Materials and methods

### Cell culture

MDA-MB-231, T47D, and HEK293T cells were obtained from the American Type Culture Collection (Manassas, VA, USA) and cultured in Dulbecco’s modified Eagle’s medium (DMEM) or RPMI-1640 with 10% fetal bovine serum (FBS) and 1% penicillin/streptomycin. All cells were maintained at 37 °C with 5% CO_2_. All cell lines were mycoplasma-free.

### Plasmids

Plasmids containing truncated human UBE3B or VHL genes were constructed by PCR amplification. cFuGW-Flag or cFuGW-Myc served as the lentiviral vectors for these genes. For protein purification, pGEX-6P-1 was used as carrier vectors for full-length VHL. VHL catalytically dead mutant (Y112H) and UBE3B with different lysine residue mutations were constructed by site-directed mutagenesis PCR. shRNA (#2 primer: 5′- CCCACTAAATACTACCAAATA−3′) against VHL were cloned into the pLKO.1 lentiviral vector. All plasmids were confirmed by DNA sequencing. Other plasmids were described previously [7, 33].

### Antibodies and reagents

Anti-UBE3B (NBP1-54950) was obtained from Novus (Centennial, CO, USA). MG132 (ab141003), Anti-UBE3B (ab83834), anti-ubiquitin (linkage-specific K63, ab179424), anti-hydroxyproline antibody (ab37067), and anti-ubiquitin (linkage-specific K48, ab140601) were obtained from Abcam (Cambridge, MA, USA). Normal mouse IgG antibody (sc-2025), anti-GST (sc-138), anti-VHL (sc-17780), anti-Endomucin (sc-65495), and anti-His (sc-8036) were obtained from Santa Cruz (Dallas, TX, USA). Anti-Flag-M2 (F1804) was obtained from Sigma (Saint Louis, USA). Anti-HA (51064-2-AP), anti-β-actin (66009-1-Ig), anti-Myc (16286-1-AP), anti-Ki-67 (27309-1-AP), HRP-conjugated affinipure goat anti-mouse secondary antibody (SA00001-1), and HRP-conjugated affinipure goat anti-rabbit secondary antibody (SA00001-2) were obtained from Proteintech (Wuhan, Hubei, China). Anti-HIF-1α (A300-286A) and anti-HIF-2α (A700-003) were obtained from Bethyl (Montgomery, TX, USA). DMOG (D3695), Cycloheximide (CHX, 2112S), Anti-VHL (2738), anti-HIF-2α (59973 S), anti-ubiquitin (3936S), anti-cleaved caspase-3 (9661S) and normal rabbit IgG antibody (2729) were obtained from Cell Signaling Technology (Danvers, MA, USA).

### Lentivirus production

Lentivirus was generated according to a previous study [7, 57, 58]. Briefly, the transducing vector and packaging vectors pMD2.G and psPAX2 were transfected into HEK293T cells using Polyjet (SignaGen, Frederick, MD, USA) per the manufacturer’s protocol. 48 h after transfection, virus particles were harvested, condensed, and transduced into breast cancer cells.

### Cell proliferation assay

Breast cancer cells were seeded into 6-well plates at a density of 2 × 10^5^ per well and cultured for 24, 48, or 72 h. The cell proliferation rate was calculated based on the number of cells at the three time points.

### Colony-formation assay

One hundred breast cancer cells were plated into 6-well plates. After 14 days, the colonies were fixed with 4% polyformaldehyde, stained with 0.5% crystal violet, and washed with PBS. Then, the number of colonies was counted.

### Cell migration and invasion assay

Breast cancer cells were plated into the upper chamber or in a Matrigel-coated upper chamber with a serum-free medium at a density of 4 × 10^4^ cells per well. The lower chamber was filled with medium containing 10% FBS. After 16 h (for migration) or 24 h (for invasion), cells on the lower surface of the transwell insert were counted by Image J after being fixed with methanol, stained with 0.5% crystal violet, and washed.

### Immunoprecipitation and western blot

Total protein was extracted using lysis buffer (10 mM Tris-HCl, 150 mM NaCl, 1 mM EDTA, 0.5% NP-40, pH 7.4) with a protease inhibitor cocktail. To quantify protein levels, 1% (for exogenous proteins) and 5% (for endogenous proteins) of the total proteins were used as whole-cell lysate (WCL). The remaining cell lysates were incubated overnight with the indicated primary antibody and protein G/A magnetic beads (Bio-Rad) at 4 °C. Subsequently, the beads were washed three times with lysis buffer, and immunoprecipitates were eluted by boiling for six minutes. The interaction of proteins was then detected by SDS-PAGE.

### In vivo ubiquitination assay

In vivo ubiquitination assays were performed as described previously [7, 58]. Briefly, cells with ectopic Flag-VHL expression or VHL ablation were treated with 10 μM MG132 for 6 h. Endogenous UBE3B was precipitated by anti-UBE3B antibody and then subjected to an immunoblotting assay to determine its ubiquitination levels using anti-ubiquitin (linkage-specific K63, ab179424) or anti-ubiquitin (linkage-specific K48, ab140601) antibody.

### In vitro ubiquitylation assay

In vitro ubiquitination assays were performed as described previously [7, 57]. In brief, immunoprecipitated GST-VHL protein and 0.5 μg purified His-UBE3B were incubated in ubiquitination buffer (50 mM Tris, pH 7.4, 5 mM MgCl_2_, 2 mM dithiothreitol, 2 mM ATP) with 50 ng human recombinant E1 Uba1 (Boston Biochem, Cambridge, MA, USA), 100 ng E2 Ubc5a (Boston Biochem), 3 μg Ub (Boston Biochem) for 2 h at 37 °C. Then, an anti-ubiquitin (linkage-specific K48, ab140601) antibody was used to evaluate the ubiquitination level of UBE3B.

### Pull-down assay

Full-length GST-HIF-2α as well as His-UBE3B were induced in *E. Coli* BL21 cells. The cell lysate was incubated with glutathione-Sepharose 4B (GE Healthcare, Chicago, IL, USA) or Ni-NTA resin (GE Healthcare), respectively, to obtain purified proteins. Purified GST-HIF-2α were incubated with purified His-UBE3B in equal amounts, and beads were added for co-incubation. Subsequently, beads were washed extensively five times before being boiled, and western blotting was performed to analyze the proteins bound to the beads.

### Luciferase reporter assay

Luciferase assays were performed according to the previous report [59]. Briefly, breast cancer cells were transfected with HIF luciferase reporter vector which contains 2× HREs from the human *VEGFA* gene and control reporter plasmid pSV-Renilla. 24 h later, cells were harvested for luciferase activity assay using the Dual Luciferase Reporter Assay Kit (Promega, Madison, WI, USA) as per the manufacturer’s instruction.

### RT-qPCR

Total RNA was isolated from cultured cells using Trizol reagent (Invitrogen, Waltham, MA, USA) and treated with DNase I (Thermo Fisher Scientific, Waltham, MA, USA). RT-qPCR assays were performed according to the standard protocol [7, 59]. The following primer pairs were used for RT-qPCR: *UBE3B* (5′- CTGTACCTCACGATGCTTGTC-3′ and 5′- TGCTGGTTGAGATGTCCCATTA-3′), *VEGFA* (5’-CTTGCCTTGCTGCTCTAC-3’ and 5’-TGGCTTGAAGATGTACTCG-3’), and *CCND1* (5’-GCTGCGAAGTGGAAACCATC-3’ and 5’-CCTCCTTCTGCACACATTTGAA-3’).

### Animal studies

Animal studies were approved by the Institutional Animal Care and Use Committee at Shandong Normal University. Female nonobese diabetic-severe combined immunodeficiency (NOD-SCID) mice (6–8 weeks old) were kept in a specific pathogen-free (SPF) facility for xenograft assays. 2 × 10^6^ of MDA-MB-231 or 6 × 10^6^ of T47D cells resuspended in 50 μL PBS were mixed with the same volume of Matrigel (Corning, Corning, NY, USA) and injected into the second left mammary fat pad of female NOD-SCID mice. After 10–13 days, the tumor was palpable, and we measured the tumor volume every three days with a caliper. The formula was adopted: V = 0.52 × L × H × W (V: volume, L: length, H: height, W: width). When the volume of primary tumors reached about 1500 mm^3^, we removed them and weighed them for quantitative analysis, and photographed them simultaneously. To study the metastasis of the tumor, we removed the lungs of mice for relevant analysis, including hematoxylin-eosin (H&E) staining of the left lung followed by PBS infusion, 0.5% agarose aeration, formalin fixation, and paraffin embedding. For the right lung, we extracted genomic DNA and performed qPCR analysis of human *HK2* (primers: 5′-CCAGTTCATTCACATCATCAG-3′ and 5′-CTTACACGAGGTCACATAGC-3′). For the tail vein injection, 1 × 10^6^ of MDA-MB-231 cells resuspended in 100 μL PBS were slowly injected into the tail vein of female NOD-SCID mice. After four weeks, the lungs were harvested for subsequent H&E staining and qPCR as described above.

### Immunohistochemistry (IHC)

IHC assays were performed according to the previous description [60, 61]. Briefly, tumors were excised from mice, fixed in neutral buffered formalin, paraffin-embedded and sectioned. Tissue ribbons were placed onto slides. The slides were baked, deparaffinized, and hydrated, followed by antigen retrieval in Citrate Antigen Retrieval solution. Endogenous peroxidase activity was blocked with 3% peroxide-methanol, and non-specific staining was then blocked with 10% goat serum. The tissues were then incubated with primary antibodies overnight at 4 °C and then HRP-conjugated secondary antibodies for 1 h at room temperature. The following primary antibodies were used: Ki-67 (1: 5000), cleaved caspase-3 (1:1500), VHL (1:500), Endomucin (1:2000), and UBE3B (1:150). The staining was visualized using the Pannoramic MiDi II slide scanner (3D Histech, Budapest, Hungary). The number of cleaved caspase-3-positive cells or Ki-67-positive cells per field was counted manually.

### Immunostaining

IHC assays were performed according to the previous description [62]. In brief, formalin-fixed paraffin-embedded breast cancer tissue arrays were baked, deparaffinized, and hydrated, followed by antigen retrieval in Citrate Antigen Retrieval solution. The tissues were then incubated sequentially with 0.3% Triton X-100, 10% goat serum, primary antibodies, Alexa Fluor 488- or Alexa Fluor 594-conjugated secondary antibodies, and DAPI, then rinsed in PBS and mounted with an antifade mounting solution. Immunofluorescence images were captured with Leica TCS SPE confocal microscope (Leica).

### Statistical analysis

At least 3 samples prepared from independent experiments were used to ensure adequate power for all studies. Statistical analysis was carried out by one-way or two-way ANOVA with multiple testing correlations within multiple groups. Data were presented as mean ± SD. *p* < 0.05 is considered significant.

### Supplementary information

Supplemental Figures and Table

Original Data

## Data Availability

The human Protein Atlas (HPA, https://www.proteinatlas.org/) were utilized for UBE3B protein levels analysis in human breast tumors. UBE3B mutations datasets were downloaded from Integrative Onco Genomics (IntOGen, https://www.intogen.org/search) and cBio Cancer Genomics Portal (cBioPortal, https://www.cbioportal.org/). All data generated or analyzed during this study are included in this article and its supplementary information files. Any additional data presented in this paper are available from the corresponding author upon request.
