# Supplementary material for: VHL suppresses UBE3B-mediated breast tumor growth and metastasis
Source: Cell Death Dis. 2024 Jun 24;15(6):446. doi: 10.1038/s41419-024-06844-x (PMC11196597; doi:10.1038/s41419-024-06844-x)
Supplement: Supplementary file 1 — Supplemental Figures and Table [file 41419_2024_6844_MOESM1_ESM.pdf]

Supplemental Figures and Table

VHL suppresses UBE3B-mediated breast tumor growth and metastasis

Shuo Wang[1], Huiyan Li[1], Xiong Liu[2], Tingting Yin[1], Tingru Li[1], Miaomiao Zheng[1], Min Liu[1],

Xiaoqian Meng[1], Jun Zhou[1], Yijie Wang[1,*], and Yan Chen[1,2,*]

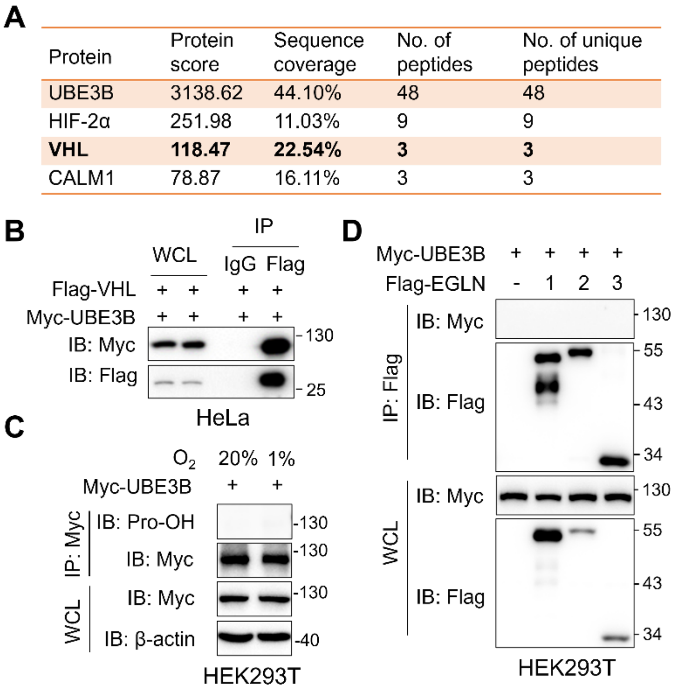

Fig. S1 UBE3B interacts with VHL independent of EGLNs. A Representatives of potential UBE3B-interacting proteins. **B** HeLa cells transfected with vectors expressing Flag-VHL and Myc-UBE3B were subjected to Co-IP assays using IgG or anti-Flag antibody. **C** MDA-MB-231 cells expressing Myc-UBE3B were cultured under normoxia or hypoxia for 24 hours, then the hydroxylation of immunoprecipitated Myc-UBE3B was detected with anti-hydroxyproline antibody. **D** Co-IP of exogenous Myc-UBE3B with Flag-EGLN1, Flag-EGLN2, or Flag-EGLN3 in HEK293T cells. WCL, whole cell lysate.

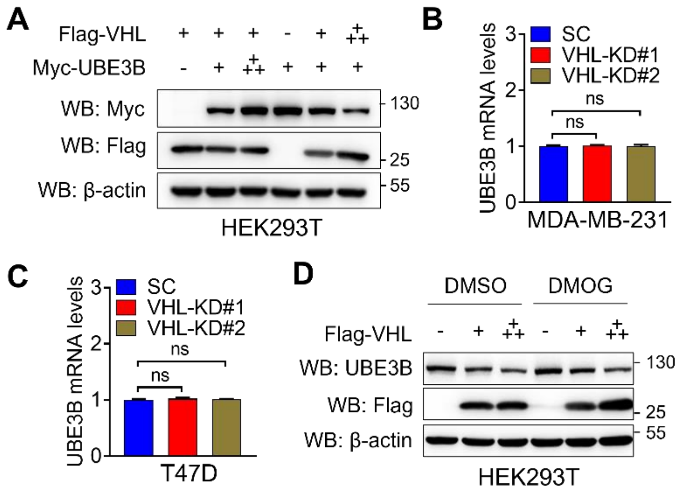

Fig. S2 UBE3B knockdown suppresses the oncogenic properties of breast cancer cells. A The impact of Myc-UBE3B on Flag-VHL stability and vice versa. **B, C** UBE3B's mRNA levels in scrambled shRNA (SC) and VHL knockdown (KD) MDA-MB-231 cells (B) or T47D cells (C) were determined by RT-qPCR. **D** The protein levels of endogenous UBE3B were analyzed in Flag-VHL-expressing MDA-MB-231 cells treated with either DMSO or 1 mM DMOG.

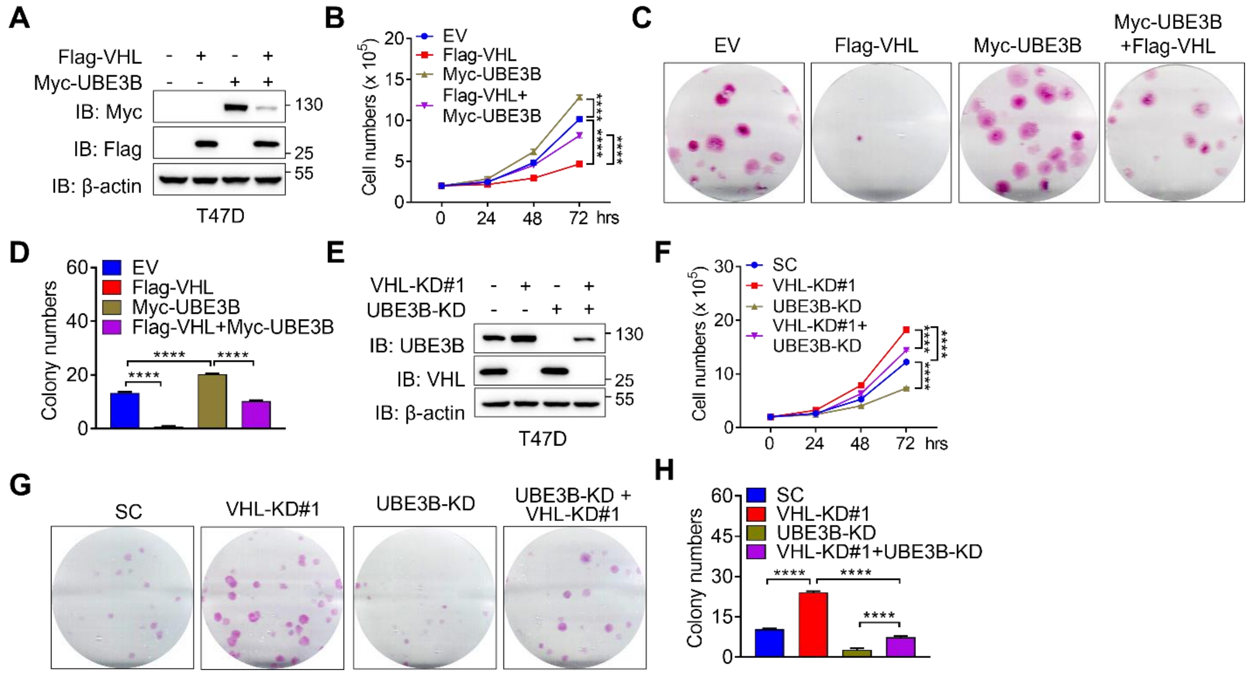

Fig. S3 VHL inhibits the oncogenic potential of breast cancer cells by suppressing UBE3B. A Protein levels of Flag-VHL and Myc-UBE3B in T47D cells stably expressing Flag-VHL or Myc-UBE3B. **B** The cell proliferation rate was measured in the indicated T47D cells (mean ± SD, n=3). ****P<0.0001, by 2-way ANOVA Tukey's multiple comparisons test. hrs, hours. **C, D** Colony formation of the indicated T47D cells, with representative images from three experiments were shown in (C). Colony numbers (D) were quantified (mean ± SD, n=3). ****P<0.0001, by 1-way ANOVA Tukey's multiple comparisons test. **E** Protein levels of VHL and UBE3B were detected in control, VHL-KD#1, UBE3B-KD, and VHL- and UBE3B-double knockdown (VHL-KD#1 + UBE3B-KD) T47D cells. **F** Proliferation rate of SC, VHL-KD#1, UBE3B-KD, and VHL-KD#1 + UBE3B-KD T47D cells (mean ± SD, n=3). ****P<0.0001, by 2-way ANOVA Tukey's multiple comparisons test. hrs, hours. **G, H** Colony formation were performed in control, VHL-KD#1, UBE3B-KD, and VHL-KD#1 + UBE3B-KD MDA-MB-231 cells, and the representative images from three experiments were shown (G). Colony numbers (H) were quantified (mean ± SD, n=3). ****P<0.0001, by 1-way ANOVA Tukey's multiple comparisons test.

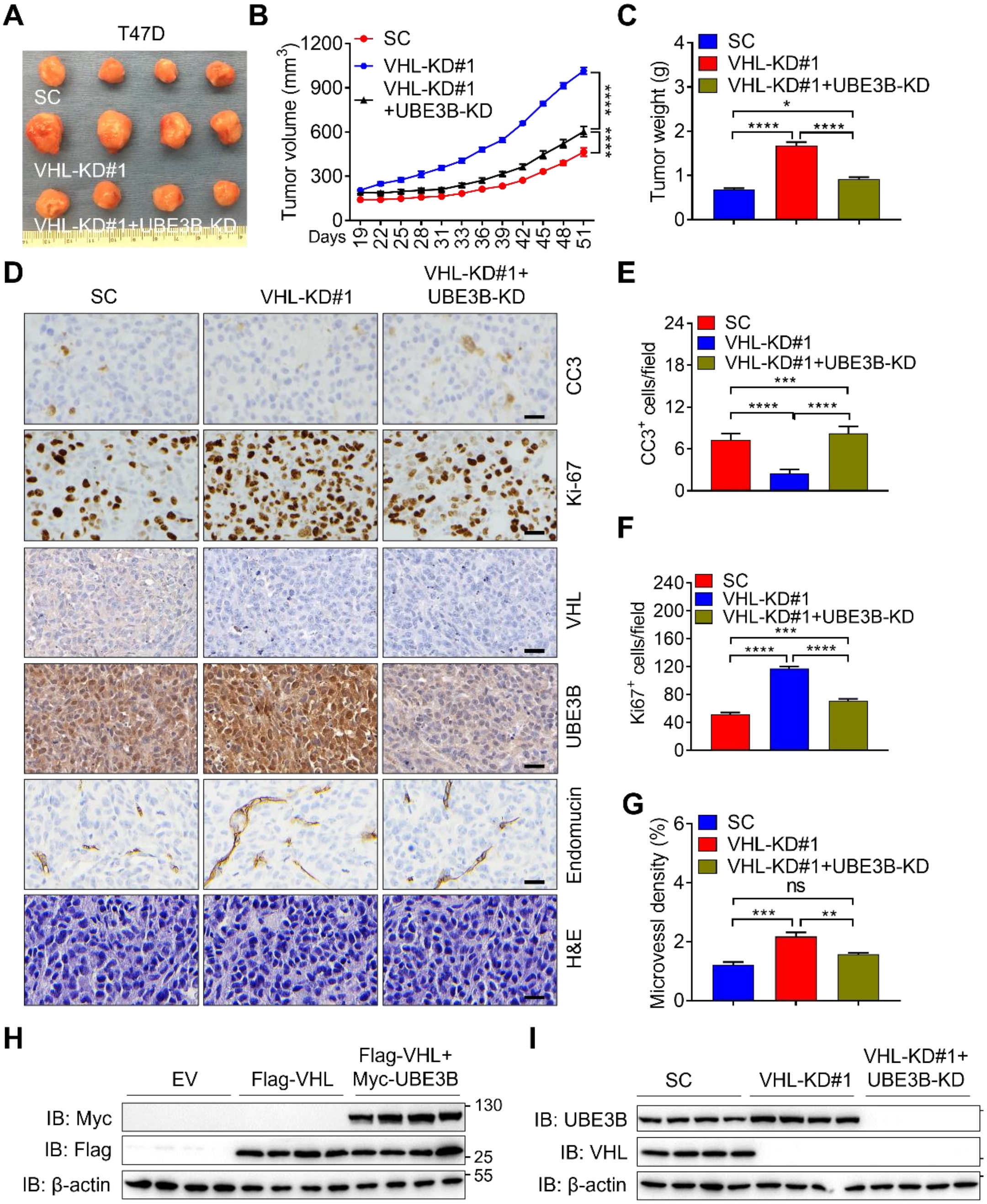

Fig. S4 VHL functions via UBE3B in regulation of breast tumor growth and metastasis. A-C Tumor images (A), tumor growth curves (B) and tumor weight (C) of mice with orthotopic implantation of SC, VHL-KD#1, and VHL-KD#1 +UBE3B-KD T47D cells into the mammary fat pad (mean ± SD, n=4). ****P<0.0001, by 2-way ANOVA Tukey's multiple comparisons test (B) or 1-way ANOVA Tukey's multiple comparisons test (C). **D-G** Representative H&E and immunohistochemical staining of Ki-67, CC3 (cleaved caspase 3), and Endomucin in primary

tumors (D). Scale bars: 100 μm. Ki-67-positive cell numbers (E), CC3-positive cell numbers (F), and microvessel density (G) were quantified. (mean ± SD, n=4). ***P<0.001, ****P<0.0001, by 1-way ANOVA Dunnett's multiple comparisons test. **H, I** Protein levels of UBE3B and VHL in MDA-MB-231 tumors (H) and T47D tumors (I).

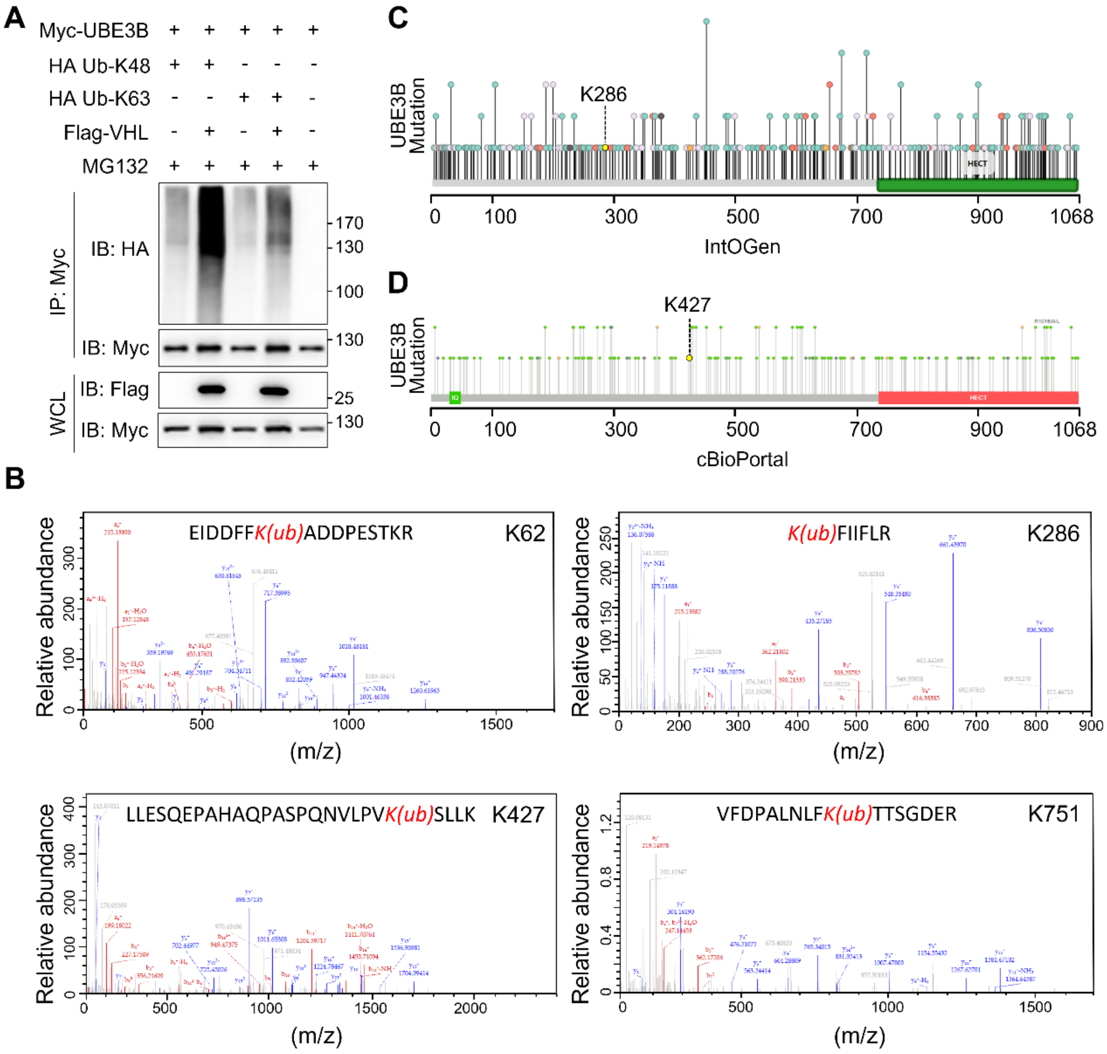

Fig. S5 Lysine residues 286 and 427 of UBE3B are key ubiquitination sites by VHL. A Ubiquitination of Myc-UBE3B in HEK293T cells with or without Flag-VHL. B The mass spectra of ubiquitinated UBE3B peptides. C, D Mutations of K286 and K427 have been identified in different tumors by analyzing datasets from Integrative Onco Genomics (IntOGen) and cBio Cancer Genomics Portal (cBioPortal).

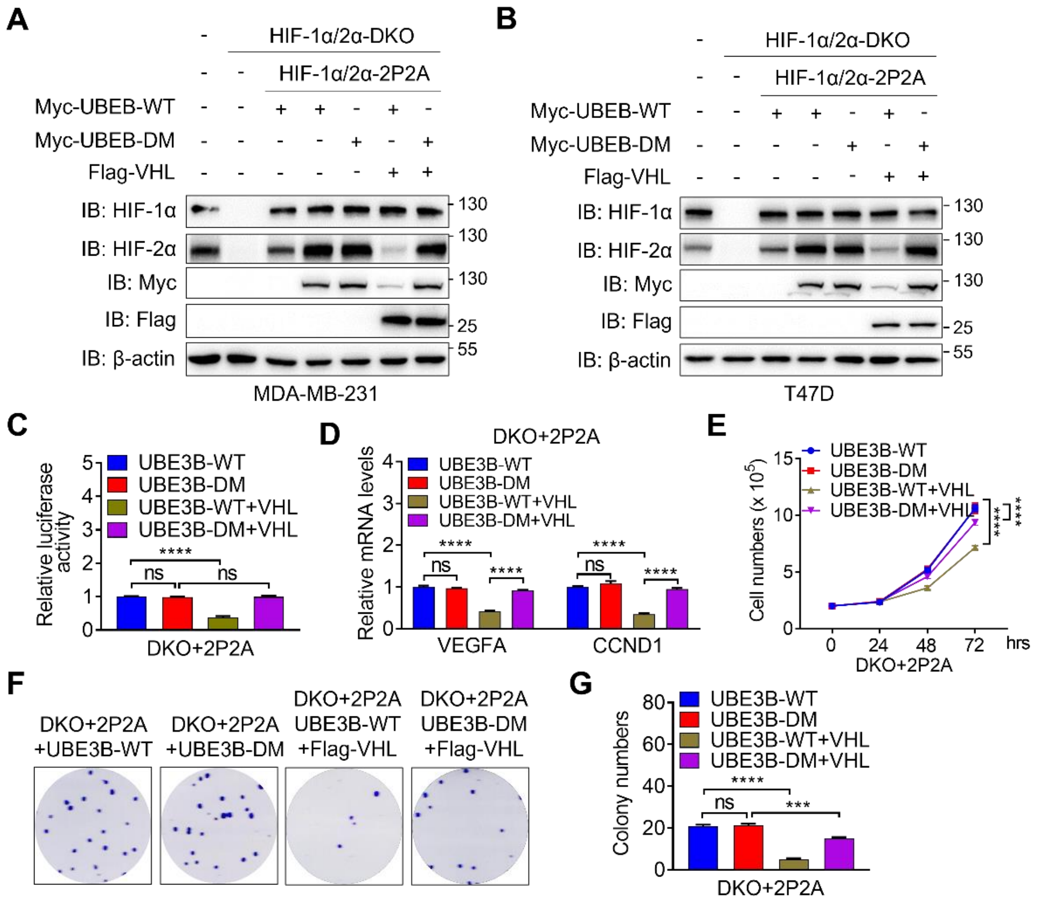

Fig. S6 VHL suppresses the oncogenic potential of breast cancer cells by ubiquitinating UBE3B at lysine residues 286 and 427. A, B Protein levels of HIF-1α, HIF-2α, Myc-UBE3B and Flag-VHL in control, HIF-1α/HIF-2α double knockout (DKO), DKO + HIF-1α/HIF-2α-2P2A (HIF-1α-P402/564A [HIF-1α-P2A] and HIF-2α-P405/531A [HIF-2α-P2A]), DKO + 2P2A + Myc-UBE3B-WT, DKO + 2P2A + Myc-UBE3B-K286/427R (DM), DKO + 2P2A + Myc-UBE3B-WT + Flag-VHL, and DKO + 2P2A + Myc-UBE3B-DM + Flag-VHL MDA-MB-231(A) and T47D (B) cells. **C, D** Luciferase activity (C) and mRNA levels of VEGFA and CCND1 (D) were measured in DKO + 2P2A + Myc-UBE3B-WT, DKO + 2P2A + Myc-UBE3B-DM, DKO + 2P2A + Myc-UBE3B-WT + Flag-VHL, and DKO + 2P2A + Myc-UBE3B-DM + Flag-VHL T47D cells (mean ± SD, n=3). ****P<0.0001, by 1-way ANOVA Tukey's multiple comparisons test. **E-G** Proliferation (E) and colony formation (F, G) were performed using DKO + 2P2A + Myc-UBE3B-WT, DKO + 2P2A + Myc-UBE3B-DM, DKO + 2P2A + Myc-UBE3B-WT + Flag-VHL, and DKO

+ 2P2A + Myc-UBE3B-DM + Flag-VHL T47D cells (mean ± SD, n=3). ****P<0.0001, by 2-way ANOVA Tukey's multiple comparisons test (D, E) or 1-way ANOVA Tukey's multiple comparisons test (C, G). ns, no significance.

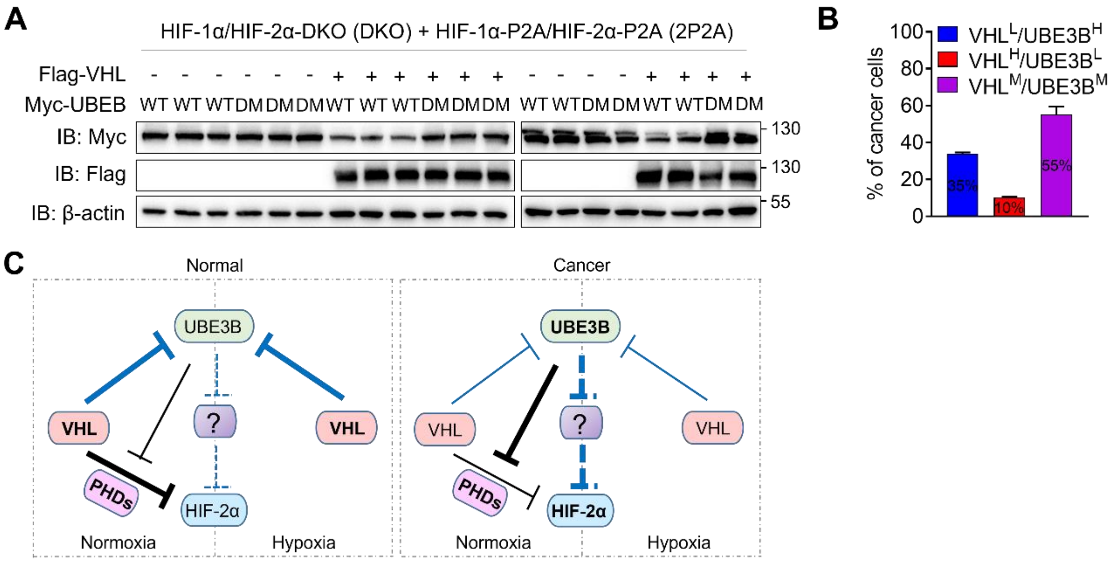

Fig. S7 VHL suppresses breast tumor growth and metastasis through ubiquitinating UBE3B at lysine residues 286 and 427. A Myc-UBE3B and Flag-VHL protein levels in T47D breast tumor tissues, as described in Fig. 7A. **B** Percentage of cancer cells that displayed a negative correlation between the protein levels of UBE3B and VHL. H, high; L, low; M, medium. **C** Schematic model illustrating the regulatory network among VHL, UBE3B, and HIF-2α in breast cancer cells. VHL physically interacts with UBE3B and HIF-2α, promoting their proteasomal degradation. Conversely, UBE3B counteracts the degradation of HIF-2α by VHL and other unknown E3 ligase(s) (indicated by question mark) in both hypoxia and normoxia. In normal breast cells, elevated VHL protein levels keep UBE3B and HIF-2α at lower levels, maintaining basic cellular functions; In breast cancer cells, reduced VHL protein levels lead to the attenuation of both VHL- and other unknown E3 ligase(s)-mediated HIF-2α degradation. Notably, VHL-mediated UBE3B degradation and UBE3B's activity remain unaffected by oxygen levels.

Table S1 Identification of UBE3B-interating proteins by LC-MS/MS analysis

Accession	Protein names	Gene names	MW [kDa]	Protein score	Sequence coverage (%)	# Unique Peptides	# Peptides	# PSMs
P35579	Myosin-9	MYH9	226.39	5356.58	53.37	83	99	172
Q7Z3V4	Ubiquitin-protein ligase E3B	UBE3B	123.02	3138.62	44.10	48	48	128
P35580	Myosin-10	MYH10	228.86	3042.51	45.65	65	78	105
Q9UM54	Unconventional myosin-VI	MYO6	149.60	1129.55	26.20	29	29	40
P09874	Poly [ADP-ribose] polymerase 1	PARP1	113.01	1041.67	31.56	31	31	42
P63261	Actin, cytoplasmic 2	ACTG1	41.77	1029.96	66.67	10	18	36
P04264	Keratin, type II cytoskeletal 1	KRT1	66.00	950.81	43.01	18	22	28
P52272	Heterogeneous nuclear ribonucleoprotein M	HNRNPM	77.46	941.54	36.85	22	22	31
Q7Z406	Myosin-14	MYH14	227.73	929.68	17.29	20	29	35
P35527	Keratin, type I cytoskeletal 9	KRT9	62.03	880.23	41.41	17	17	23
Q16891	MICOS complex subunit MIC60	IMMT	83.63	837.04	38.39	24	24	32
Q9UQ35	Serine/arginine repetitive matrix protein 2	SRRM2	299.44	725.63	12.75	23	23	27
P06748	Nucleophosmin	NPM1	32.56	720.23	30.61	7	7	15
O14950	Myosin regulatory light chain 12B	MYL12B	19.77	658.74	50.58	8	8	19
P13645	Keratin, type I cytoskeletal 10	KRT10	58.79	641.60	32.71	16	18	19
P39023	60S ribosomal protein L3	RPL3	46.08	620.25	36.23	14	14	23
P61247	40S ribosomal protein S3a	RPS3A	29.93	606.00	44.70	11	11	19
P17844	Probable ATP-dependent RNA helicase DDX5	DDX5	69.11	602.66	33.55	13	17	23
Q9NYF8	Bcl-2-associated transcription factor 1	BCLAF1	106.06	583.35	22.17	15	15	21
P35908	Keratin, type II cytoskeletal 2 epidermal	KRT2	65.39	579.16	33.33	14	19	22
P11142	Heat shock cognate 71 kDa protein	HSPA8	70.85	561.53	23.37	9	11	15
P19338	Nucleolin	NCL	76.568	530.4834	23.38028169	15	15	17
P05387	60S acidic ribosomal protein P2	RPLP2	11.66	526.77	85.22	7	7	11

Q9BQG0	Myb-binding protein 1A	MYBBP1A	148.76	521.07	13.25	16	16	18
Q9P2E9	Ribosome-binding protein 1	RRBP1	152.37	515.11	15.53	16	16	17
Q02878	60S ribosomal protein L6	RPL6	32.71	512.53	39.58	14	14	20
Q92841	Probable ATP-dependent RNA helicase DDX17	DDX17	80.22	505.59	27.16	13	17	20
P05388	60S acidic ribosomal protein P0	RPLP0	34.25	504.05	49.84	12	12	17
P06899	Histone H2B type 1-J	H2BC11	13.90	494.63	41.27	2	6	15
Q9NR30	Nucleolar RNA helicase 2	DDX21	87.29	492.34	25.16	16	17	22
O60814	Histone H2B type 1-K	H2BC12	13.88	483.61	41.27	1	5	14
P09651	Heterogeneous nuclear ribonucleoprotein A1	HNRNPA1	38.72	474.39	31.99	7	9	13
P62805	Histone H4	H4C1	11.36	468.32	47.57	6	6	15
O94832	Unconventional myosin-Id	MYO1D	116.13	464.16	28.33	25	25	27
P11940	Polyadenylate-binding protein 1	PABPC1	70.63	461.58	29.56	12	18	22
P08238	Heat shock protein HSP 90-beta	HSP90AB1	83.21	453.47	28.59	9	17	20
Q8IWS0	PHD finger protein 6	PHF6	41.26	447.14	22.74	7	7	12
Q9NZI8	nsulin-like growth factor 2 mRNA-binding protein	IGF2BP1	63.44	442.98	22.70	9	10	11
P68032	Actin, alpha cardiac muscle 1	ACTC1	41.99	441.68	26.26	1	9	22
Q9Y2W1	Thyroid hormone receptor-associated protein 3	THRAP3	108.60	440.70	23.87	20	20	22
P23396	40S ribosomal protein S3	RPS3	26.67	439.83	67.08	15	15	20
O00571	ATP-dependent RNA helicase DDX3X	DDX3X	73.20	437.10	26.44	12	13	15
P62263	40S ribosomal protein S14	RPS14	16.26	437.07	36.42	5	5	10
Q08J23	RNA cytosine C(5)-methyltransferase NSUN2	NSUN2	86.42	418.13	22.16	14	14	18
Q8IYB3	Serine/arginine repetitive matrix protein 1	SRRM1	102.27	398.36	15.60	9	9	11
Q9UKV3	optotic chromatin condensation inducer in the nucl	ACIN1	151.77	394.78	13.50	14	14	15
Q08211	ATP-dependent RNA helicase A	DHX9	140.87	388.65	14.88	18	18	22
P32969	60S ribosomal protein L9	RPL9	21.85	385.68	53.65	8	8	13
P08708	40S ribosomal protein S17	RPS17	15.54	380.65	54.81	5	5	11
Q9BVP2	Guanine nucleotide-binding protein-like 3	GNL3	61.95	377.55	24.59	10	11	13

Q6PKG0	La-related protein 1	LARP1	123.43	374.88	11.86	9	9	11
P62753	40S ribosomal protein S6	RPS6	28.66	370.49	29.72	9	9	13
O75643	J5 small nuclear ribonucleoprotein 200 kDa helicas	SNRNP200	244.35	366.96	8.19	15	15	16
P62701	40S ribosomal protein S4, X isoform	RPS4X	29.58	366.73	48.67	14	14	21
O00159	Unconventional myosin-Ic	MYO1C	121.61	358.14	21.17	18	18	19
P62424	60S ribosomal protein L7a	RPL7A	29.98	333.35	41.35	13	13	15
Q01130	Serine/arginine-rich splicing factor 2	SRSF2	25.46	323.69	33.48	5	7	10
O75533	Splicing factor 3B subunit 1	SF3B1	145.74	318.45	11.66	11	11	14
Q9NVI7	ATPase family AAA domain-containing protein 3A	ATAD3A	71.33	317.48	23.50	4	13	13
P68363	Tubulin alpha-1B chain	TUBA1B	50.12	309.67	25.94	9	9	11
P0DMV8	Heat shock 70 kDa protein 1A	HSPA1A	70.01	309.34	22.15	9	10	11
P04843	phosphooligosaccharide--protein glycosyltransferas	RPN1	68.53	308.00	18.45	10	10	10
P62888	60S ribosomal protein L30	RPL30	12.78	300.47	59.13	7	7	11
Q6P2Q9	Pre-mRNA-processing-splicing factor 8	PRPF8	273.43	299.66	6.85	16	16	16
P62829	60S ribosomal protein L23	RPL23	14.86	296.78	56.43	6	6	11
Q86V81	THO complex subunit 4	ALYREF	26.87	293.17	29.96	6	6	9
P26373	60S ribosomal protein L13	RPL13	24.25	293.11	34.60	8	8	9
P25398	40S ribosomal protein S12	RPS12	14.51	284.20	41.67	4	4	8
P78527	DNA-dependent protein kinase catalytic subunit	PRKDC	468.79	283.77	2.64	10	10	11
Q07020	60S ribosomal protein L18	RPL18	21.62	281.42	39.36	7	7	8
P46781	40S ribosomal protein S9	RPS9	22.58	279.10	37.11	11	11	12
Q9UJV9	Probable ATP-dependent RNA helicase DDX41	DDX41	69.79	278.72	20.42	10	10	11
P60660	Myosin light polypeptide 6	MYL6	16.92	278.67	54.30	5	7	11
P62081	40S ribosomal protein S7	RPS7	22.11	278.56	51.55	7	7	11
P38159	RNA-binding motif protein, X chromosome	RBMX	42.31	275.98	31.46	12	12	12
P30050	60S ribosomal protein L12	RPL12	17.81	274.51	42.42	6	6	8
P46777	60S ribosomal protein L5	RPL5	34.34	273.09	20.88	7	7	13

P10809	60 kDa heat shock protein, mitochondrial	HSPD1	61.02	270.64	16.06	7	7	8
P62269	40S ribosomal protein S18	RPS18	17.71	270.42	47.37	9	9	10
Q7L2E3	ATP-dependent RNA helicase DHX30	DHX30	133.86	269.98	10.13	11	11	12
O43143	NA-splicing factor ATP-dependent RNA helicase l	DHX15	90.88	269.57	11.32	8	8	11
P22626	Heterogeneous nuclear ribonucleoproteins A2/B1	HNRNPA2B1	37.41	267.76	25.50	7	9	13
Q00839	Heterogeneous nuclear ribonucleoprotein U	HNRNPU	90.53	263.73	16.12	10	10	13
P26368	Splicing factor U2AF 65 kDa subunit	U2AF2	53.47	262.49	19.58	5	5	10
Q9UHX1	Poly(U)-binding-splicing factor PUF60	PUF60	59.84	261.39	19.32	6	6	10
P07900	Heat shock protein HSP 90-alpha	HSP90AA1	84.61	261.03	15.16	2	10	12
P06396	Gelsolin	GSN	85.64	259.32	11.00	7	7	7
P62244	40S ribosomal protein S15a	RPS15A	14.83	257.95	51.54	6	6	8
P18621	60S ribosomal protein L17	RPL17	21.38	253.91	35.33	7	7	12
P67809	Y-box-binding protein 1	YBX1	35.90	253.49	28.70	5	6	7
Q14498	RNA-binding protein 39	RBM39	59.34	252.52	23.02	9	9	10
Q9BZE4	Nucleolar GTP-binding protein 1	GTPBP4	73.92	252.26	20.35	11	11	11
Q99814	Endothelial PAS domain-containing protein 1	EPAS1	96.40	251.98	11.03	9	9	10
P61513	60S ribosomal protein L37a	RPL37A	10.27	250.98	45.65	5	5	10
Q96HS1	e/threonine-protein phosphatase PGAM5, mitochoi	PGAM5	31.99	249.32	38.06	12	12	13
O14654	Insulin receptor substrate 4	IRS4	133.69	248.75	11.06	11	11	13
Q07955	Serine/arginine-rich splicing factor 1	SRSF1	27.73	245.85	39.52	10	10	12
Q6P5R6	60S ribosomal protein L22-like 1	RPL22L1	14.60	244.85	25.41	3	3	5
Q9UN86	Ras GTPase-activating protein-binding protein 2	G3BP2	54.09	244.68	22.82	6	7	11
P15880	40S ribosomal protein S2	RPS2	31.31	242.59	38.91	11	11	12
Q14974	Importin subunit beta-1	KPNB1	97.11	235.28	13.13	8	8	9
O00567	Nucleolar protein 56	NOP56	66.01	231.18	16.33	6	6	9
Q08170	Serine/arginine-rich splicing factor 4	SRSF4	56.65	229.28	12.15	3	6	6
P51116	agile X mental retardation syndrome-related protei	FXR2	74.18	228.96	10.70	4	5	6

P51991	Heterogeneous nuclear ribonucleoprotein A3	HNRNPA3	39.57	227.86	15.08	4	5	7
P83731	60S ribosomal protein L24	RPL24	17.77	224.64	36.31	6	6	8
Q9H0A0	RNA cytidine acetyltransferase	NAT10	115.66	224.53	11.12	8	9	9
P36578	60S ribosomal protein L4	RPL4	47.67	224.46	25.29	9	9	9
Q9BYG3	:I67 FHA domain-interacting nucleolar phosphopro	NIFK	34.20	223.98	24.91	5	5	6
P51114	agile X mental retardation syndrome-related protei	FXR1	69.68	222.78	11.11	5	6	7
P63173	60S ribosomal protein L38	RPL38	8.21	221.99	32.86	5	5	7
O76021	Ribosomal L1 domain-containing protein 1	RSL1D1	54.94	221.88	14.08	5	5	6
Q9Y4I1	Unconventional myosin-Va	MYO5A	215.27	218.79	5.98	10	10	11
Q13283	Ras GTPase-activating protein-binding protein 1	G3BP1	52.13	218.78	20.17	6	7	11
P40429	60S ribosomal protein L13a	RPL13A	23.56	216.96	45.81	11	11	12
Q00325	Phosphate carrier protein, mitochondrial	SLC25A3	40.07	212.90	12.71	4	4	5
P52597	Heterogeneous nuclear ribonucleoprotein F	HNRNPF	45.64	211.35	10.36	1	3	6
P61254	60S ribosomal protein L26	RPL26	17.25	210.79	49.66	2	11	12
Q13310	Polyadenylate-binding protein 4	PABPC4	70.74	209.98	13.51	1	7	8
O60841	Eukaryotic translation initiation factor 5B	EIF5B	138.74	208.35	11.72	10	10	10
O43290	U4/U6.U5 tri-snRNP-associated protein 1	SART1	90.20	207.47	12.50	6	6	7
P61313	60S ribosomal protein L15	RPL15	24.13	204.37	38.24	9	9	10
P62277	40S ribosomal protein S13	RPS13	17.21	203.83	29.80	5	5	6
Q13247	Serine/arginine-rich splicing factor 6	SRSF6	39.56	202.01	18.31	4	7	7
Q9Y2X3	Nucleolar protein 58	NOP58	59.54	201.71	12.85	5	5	6
Q9P035	ry-long-chain (3R)-3-hydroxyacyl-CoA dehydratas	HACD3	43.13	200.76	17.68	5	5	6
Q15717	ELAV-like protein 1	ELAVL1	36.07	199.29	11.66	3	3	3
Q15029	6 kDa U5 small nuclear ribonucleoprotein compon	EFTUD2	109.37	195.36	9.67	8	8	9
P62241	40S ribosomal protein S8	RPS8	24.19	189.38	37.02	7	7	9
Q96N67	Dedicator of cytokinesis protein 7	DOCK7	242.41	187.91	2.94	5	5	6
P07910	Heterogeneous nuclear ribonucleoproteins C1/C2	HNRNPC	33.65	187.46	29.41	7	7	9

P10412	Histone H1.4	H1-4	21.85	186.73	26.94	1	7	8
P62847	40S ribosomal protein S24	RPS24	15.41	186.35	39.85	6	6	11
P12532	Creatine kinase U-type, mitochondrial	CKMT1A	47.01	186.02	28.30	7	7	8
P62750	60S ribosomal protein L23a	RPL23A	17.68	185.18	47.44	8	8	10
P16403	Histone H1.2	H1-2	21.35	184.11	27.70	1	7	8
Q9UG63	ATP-binding cassette sub-family F member 2	ABCF2	71.25	182.80	13.64	7	7	10
P62995	Transformer-2 protein homolog beta	TRA2B	33.65	181.02	24.65	6	6	7
Q16629	Serine/arginine-rich splicing factor 7	SRSF7	27.35	181.02	28.57	6	7	10
Q14444	Caprin-1	CAPRIN1	78.32	180.80	8.32	6	6	6
P18124	60S ribosomal protein L7	RPL7	29.21	180.33	32.66	9	9	9
P27635	60S ribosomal protein L10	RPL10	24.59	180.31	22.43	5	5	7
P31943	Heterogeneous nuclear ribonucleoprotein H	HNRNPH1	49.20	180.28	14.70	3	5	7
P50402	Emerin	EMD	28.98	179.41	11.42	2	2	3
Q13243	Serine/arginine-rich splicing factor 5	SRSF5	31.25	178.65	19.12	4	5	6
Q4VCS5	Angiomotin	AMOT	118.01	177.57	6.27	6	6	7
Q5T9A4	ATPase family AAA domain-containing protein 3B	ATAD3B	72.53	176.72	16.67	1	10	10
Q92499	ATP-dependent RNA helicase DDX1	DDX1	82.38	175.08	14.73	8	8	9
Q99459	Cell division cycle 5-like protein	CDC5L	92.19	174.98	9.48	6	6	6
P62249	40S ribosomal protein S16	RPS16	16.44	174.27	32.88	5	5	7
O00422	Histone deacetylase complex subunit SAP18	SAP18	17.55	174.10	37.25	6	6	7
P61353	60S ribosomal protein L27	RPL27	15.79	173.75	47.06	6	6	7
P16402	Histone H1.3	H1-3	22.34	173.15	26.70	1	7	8
Q12905	Interleukin enhancer-binding factor 2	ILF2	43.04	172.79	14.36	4	4	5
P11388	DNA topoisomerase 2-alpha	TOP2A	174.28	171.48	5.09	4	6	6
Q9UNX3	60S ribosomal protein L26-like 1	RPL26L1	17.25	170.16	44.14	1	10	11
Q9Y3Y2	Chromatin target of PRMT1 protein	CHTOP	26.38	169.37	10.48	2	2	3
P84098	60S ribosomal protein L19	RPL19	23.45	169.26	30.61	6	6	8

P08865	40S ribosomal protein SA	RPSA	32.83	169.11	15.25	3	3	3
P05023	dium/potassium-transporting ATPase subunit alpha	ATP1A1	112.82	168.44	8.11	6	6	6
P30153	-protein phosphatase 2A 65 kDa regulatory subunit	PPP2R1A	65.27	167.80	10.36	4	4	5
P39019	40S ribosomal protein S19	RPS19	16.05	166.94	52.41	9	9	10
P62987	Ubiquitin-60S ribosomal protein L40	UBA52	14.72	166.75	41.41	5	5	7
P62851	40S ribosomal protein S25	RPS25	13.73	163.69	29.60	5	5	5
P62280	40S ribosomal protein S11	RPS11	18.42	163.66	44.30	9	9	10
Q02880	DNA topoisomerase 2-beta	TOP2B	183.15	163.59	3.69	3	5	5
O00425	nsulin-like growth factor 2 mRNA-binding protein	IGF2BP3	63.67	162.60	6.91	2	3	4
Q96PK6	RNA-binding protein 14	RBM14	69.45	161.79	10.91	6	6	6
P13639	Elongation factor 2	EEF2	95.28	161.75	5.94	5	5	5
P62258	14-3-3 protein epsilon	YWHAE	29.16	161.34	19.22	2	4	5
O75531	Barrier-to-autointegration factor	BANF1	10.05	161.21	34.83	3	3	5
P13647	Keratin, type II cytoskeletal 5	KRT5	62.34	161.20	12.37	4	8	8
P36542	ATP synthase subunit gamma, mitochondrial	ATP5F1C	32.98	160.07	24.50	7	7	9
Q9Y383	Putative RNA-binding protein Luc7-like 2	LUC7L2	46.49	158.31	17.35	4	6	7
Q2NL82	Pre-rRNA-processing protein TSR1 homolog	TSR1	91.75	158.23	8.96	6	6	6
P27708	CAD protein	CAD	242.83	157.50	2.74	5	5	5
P31689	DnaJ homolog subfamily A member 1	DNAJA1	44.84	156.86	16.62	5	5	6
P62917	60S ribosomal protein L8	RPL8	28.01	156.81	21.40	4	4	6
P02533	Keratin, type I cytoskeletal 14	KRT14	51.53	156.53	10.59	3	5	5
P68104	Elongation factor 1-alpha 1	EEF1A1	50.11	156.01	13.85	5	5	5
O75494	Serine/arginine-rich splicing factor 10	SRSF10	31.28	153.92	17.18	4	4	5
P62266	40S ribosomal protein S23	RPS23	15.80	151.75	23.78	4	4	7
Q96SB4	SRSF protein kinase 1	SRPK1	74.28	151.51	11.30	6	6	6
P06753	Tropomyosin alpha-3 chain	TPM3	32.93	149.20	22.81	3	7	7
P62913	60S ribosomal protein L11	RPL11	20.24	149.04	28.09	5	5	5

Q15050	Ribosome biogenesis regulatory protein homolog	RRS1	41.17	146.09	13.42	3	3	4
Q06787	Synaptic functional regulator FMR1	FMR1	71.13	145.23	8.39	5	5	6
Q9NX58	Cell growth-regulating nucleolar protein	LYAR	43.59	144.45	14.51	4	4	5
Q8NC51	asminogen activator inhibitor 1 RNA-binding prote	SERBP1	44.94	144.09	10.54	3	3	4
P62841	40S ribosomal protein S15	RPS15	17.03	143.49	31.03	5	5	7
P26599	Polypyrimidine tract-binding protein 1	PTBP1	57.19	142.66	4.33	1	1	1
P61978	Heterogeneous nuclear ribonucleoprotein K	HNRNPK	50.94	142.32	8.42	3	3	4
P27348	14-3-3 protein theta	YWHAQ	27.75	142.18	20.82	2	4	5
Q9NVN8	uanine nucleotide-binding protein-like 3-like protei	GNL3L	65.53	141.83	9.97	4	5	5
P33993	DNA replication licensing factor MCM7	MCM7	81.26	139.52	11.82	6	6	6
P11177	e dehydrogenase E1 component subunit beta, mitoc	PDHB	39.21	136.84	13.93	4	4	4
P07437	Tubulin beta chain	TUBB	49.64	136.25	10.81	4	4	5
P67936	Tropomyosin alpha-4 chain	TPM4	28.50	135.26	28.23	3	7	7
P35249	Replication factor C subunit 4	RFC4	39.66	134.04	20.39	6	6	6
Q9BU76	Multiple myeloma tumor-associated protein 2	MMTAG2	29.39	133.77	17.11	4	4	4
P60866	40S ribosomal protein S20	RPS20	13.36	133.30	19.33	2	2	3
Q13595	Transformer-2 protein homolog alpha	TRA2A	32.67	132.19	17.38	4	4	4
Q02978	Mitochondrial 2-oxoglutarate/malate carrier protei	SLC25A11	34.04	132.05	12.74	3	3	3
P63010	AP-2 complex subunit beta	AP2B1	104.49	131.59	4.91	3	3	4
O14880	Microsomal glutathione S-transferase 3	MGST3	16.51	131.51	17.76	2	2	2
P46779	60S ribosomal protein L28	RPL28	15.74	131.50	33.58	6	6	8
Q13823	Nucleolar GTP-binding protein 2	GNL2	83.60	131.49	6.70	4	4	4
Q9NNW5	WD repeat-containing protein 6	WDR6	121.65	130.38	3.75	3	3	4
Q5T3I0	G patch domain-containing protein 4	GPATCH4	50.35	129.28	17.71	7	7	9
Q8TDN6	Ribosome biogenesis protein BRX1 homolog	BRIX1	41.38	127.24	16.43	5	5	5
P52701	DNA mismatch repair protein Msh6	MSH6	152.69	126.31	3.90	4	4	5
Q5T5X7	BEN domain-containing protein 3	BEND3	94.42	126.30	9.06	6	6	6

P49411	Elongation factor Tu, mitochondrial	TUFM	49.51	125.90	9.51	3	3	4
P14649	Myosin light chain 6B	MYL6B	22.75	125.15	21.15	2	4	5
Q13435	Splicing factor 3B subunit 2	SF3B2	100.17	124.82	6.59	6	6	6
Q9UJS0	alcium-binding mitochondrial carrier protein Arala	SLC25A13	74.13	124.04	8.89	4	4	4
Q96A72	Protein mago nashi homolog 2	MAGOHB	17.27	123.55	33.78	4	4	4
Q96EY4	Translation machinery-associated protein 16	TMA16	23.85	122.12	17.24	2	2	2
P11387	DNA topoisomerase 1	TOP1	90.67	118.54	10.07	7	7	7
P40337	von Hippel-Lindau disease tumor suppressor	VHL	24.14	118.47	22.54	3	3	6
Q9UHI6	Probable ATP-dependent RNA helicase DDX20	DDX20	92.18	118.17	4.13	2	2	2
P46783	40S ribosomal protein S10	RPS10	18.89	117.16	33.33	5	5	8
Q8NE71	ATP-binding cassette sub-family F member 1	ABCF1	95.87	116.63	8.76	5	5	5
Q13151	Heterogeneous nuclear ribonucleoprotein A0	HNRNPA0	30.82	116.59	13.11	2	3	5
A1L0T0	2-hydroxyacyl-CoA lyase 2	ILVBL	67.83	115.93	3.64	1	1	2
P48634	Protein PRRC2A	PRRC2A	228.72	115.03	2.27	3	4	4
P05198	Eukaryotic translation initiation factor 2 subunit 1	EIF2S1	36.09	114.71	23.17	7	7	7
P12268	Inosine-5'-monophosphate dehydrogenase 2	IMPDH2	55.77	114.46	6.61	2	2	2
Q8NCA5	Protein FAM98A	FAM98A	55.24	114.15	2.90	1	1	1
Q8IY37	Probable ATP-dependent RNA helicase DHX37	DHX37	129.46	112.67	2.25	2	2	2
P62306	Small nuclear ribonucleoprotein F	SNRPF	9.72	112.28	15.12	1	1	2
P49916	DNA ligase 3	LIG3	112.84	112.06	6.24	6	6	6
Q02543	60S ribosomal protein L18a	RPL18A	20.75	111.23	27.27	6	6	7
Q9NYL9	Tropomodulin-3	TMOD3	39.57	110.67	9.09	2	2	2
O60506	Heterogeneous nuclear ribonucleoprotein Q	SYNCRIP	69.56	110.62	4.49	2	2	2
A0A0A0MS14	Immunoglobulin heavy variable 1-45	IGHV1-45	13.50	110.40	9.40	1	1	2
Q9BZI7	Regulator of nonsense transcripts 3B	UPF3B	57.73	110.17	10.97	5	5	5
Q92621	Nuclear pore complex protein Nup205	NUP205	227.78	109.68	1.89	3	3	3
P0DN76	Splicing factor U2AF 35 kDa subunit-like protein	U2AF1L5	27.85	109.63	13.75	3	3	3

Q13610	Periodic tryptophan protein 1 homolog	PWP1	55.79	109.42	8.78	3	3	3
P11021	Endoplasmic reticulum chaperone BiP	HSPA5	72.29	108.12	4.28	1	2	2
P22087	rRNA 2'-O-methyltransferase fibrillarin	FBL	33.76	107.03	13.40	3	3	3
Q9H583	HEAT repeat-containing protein 1	HEATR1	242.22	104.73	1.73	3	3	3
Q14690	Protein RRP5 homolog	PDCD11	208.57	103.90	1.71	3	3	3
P16615	rcoplasmic/endoplasmic reticulum calcium ATPase	ATP2A2	114.68	102.85	3.84	3	3	3
Q9HCE1	Helicase MOV-10	MOV10	113.60	102.58	6.68	6	6	6
P17812	CTP synthase 1	CTPS1	66.65	102.47	8.46	4	4	4
P50914	60S ribosomal protein L14	RPL14	23.42	101.91	25.58	6	6	6
Q07666	ontaining, RNA-binding, signal transduction-associ	KHDRBS1	48.20	101.90	7.67	2	2	4
P49368	T-complex protein 1 subunit gamma	CCT3	60.50	101.54	5.87	3	3	3
P56192	Methionine--tRNA ligase, cytoplasmic	MARS1	101.05	101.50	3.67	2	2	2
P46778	60S ribosomal protein L21	RPL21	18.55	101.09	36.25	6	6	8
Q53GS9	U4/U6.U5 tri-snRNP-associated protein 2	USP39	65.34	100.69	6.19	2	2	3
P11586	C-1-tetrahydrofolate synthase, cytoplasmic	MTHFD1	101.50	100.58	3.64	3	3	3
Q13724	Mannosyl-oligosaccharide glucosidase	MOGS	91.86	100.36	3.58	2	2	3
P47914	60S ribosomal protein L29	RPL29	17.74	100.18	14.47	2	2	2
Q14739	Delta(14)-sterol reductase LBR	LBR	70.66	99.76	4.88	2	2	2
P35250	Replication factor C subunit 2	RFC2	39.13	98.94	13.28	4	4	4
Q9UNQ2	Probable dimethyladenosine transferase	DIMT1	35.21	98.92	12.46	3	3	4
O75367	Core histone macro-H2A.1	MACROH2A1	39.59	98.46	12.63	3	3	3
P11310	m-chain specific acyl-CoA dehydrogenase, mitocho	ACADM	46.56	98.43	15.68	6	6	6
O00139	Kinesin-like protein KIF2A	KIF2A	79.91	98.17	5.10	4	4	4
P36873	hreonine-protein phosphatase PP1-gamma catalytic	PPP1CC	36.96	98.07	11.15	3	3	3
Q13573	SNW domain-containing protein 1	SNW1	61.46	97.83	6.90	2	2	3
P37108	Signal recognition particle 14 kDa protein	SRP14	14.56	97.64	19.85	2	2	2
Q15233	Ion-POU domain-containing octamer-binding protei	NONO	54.20	96.42	2.76	1	1	1

Q9Y5S9	RNA-binding protein 8A	RBM8A	19.88	96.06	25.86	2	2	2
Q8WWM7	Ataxin-2-like protein	ATXN2L	113.30	96.04	5.58	4	4	4
Q14684	Ribosomal RNA processing protein 1 homolog B	RRP1B	84.38	94.84	10.42	6	6	7
P45880	Voltage-dependent anion-selective channel protein 2	VDAC2	31.55	94.83	17.01	4	4	4
P14866	Heterogeneous nuclear ribonucleoprotein L	HNRNPL	64.09	94.60	7.47	2	2	3
Q9NQ29	Putative RNA-binding protein Luc7-like 1	LUC7L	43.70	93.86	12.94	2	4	5
Q9BRJ6	Uncharacterized protein C7orf50	C7orf50	22.07	93.86	24.23	3	3	3
P48729	Casein kinase I isoform alpha	CSNK1A1	38.89	93.45	6.23	1	1	1
P43243	Matrin-3	MATR3	94.57	93.02	5.90	3	3	3
P22695	Cytochrome b-c1 complex subunit 2, mitochondrial	UQCRC2	48.41	92.85	10.82	3	3	3
Q96T37	RNA-binding protein 15	RBM15	107.12	92.59	4.91	3	3	3
P42677	40S ribosomal protein S27	RPS27	9.46	91.97	29.76	3	3	3
Q9Y3Z3	ynucleoside triphosphate triphosphohydrolase SAM	SAMHD1	72.16	91.70	5.43	3	3	3
Q9UPN6	SR-related and CTD-associated factor 8	SCAF8	140.43	91.60	2.05	3	3	3
P04908	Histone H2A type 1-B/E	H2AC4	14.13	91.34	21.54	1	2	4
Q9Y2R4	Probable ATP-dependent RNA helicase DDX52	DDX52	67.46	91.03	5.84	2	2	4
Q13155	tRNA synthase complex-interacting multifunctiona	AIMP2	35.33	90.89	10.00	2	2	2
Q5F1R6	DnaJ homolog subfamily C member 21	DNAJC21	61.99	90.30	3.01	1	1	1
P53621	Coatomer subunit alpha	COPA	138.26	89.49	5.23	5	5	5
Q96IX5	TP synthase membrane subunit DAPIT, mitochondr	ATP5MD	6.45	89.38	25.86	1	1	1
Q13242	Serine/arginine-rich splicing factor 9	SRSF9	25.53	89.19	20.36	4	4	4
Q10570	eavage and polyadenylation specificity factor subun	CPSF1	160.78	88.89	1.18	1	1	1
P46776	60S ribosomal protein L27a	RPL27A	16.55	88.59	14.19	2	2	2
Q08945	FACT complex subunit SSRP1	SSRP1	81.02	88.39	11.14	7	7	7
Q96GQ7	Probable ATP-dependent RNA helicase DDX27	DDX27	89.78	88.04	6.66	4	4	4
P62861	40S ribosomal protein S30	FAU	6.64	87.91	32.20	3	3	4
P35268	60S ribosomal protein L22	RPL22	14.78	87.66	23.44	3	3	3

P61160	Actin-related protein 2	ACTR2	44.73	86.67	9.39	3	3	3
Q7Z2W4	Zinc finger CCCH-type antiviral protein 1	ZC3HAV1	101.37	86.49	3.33	2	2	2
O60884	DnaJ homolog subfamily A member 2	DNAJA2	45.72	86.39	10.19	3	3	4
P62316	Small nuclear ribonucleoprotein Sm D2	SNRPD2	13.52	86.32	30.51	4	4	4
Q9Y5B9	FACT complex subunit SPT16	SUPT16H	119.84	86.30	3.44	3	3	3
Q86UE4	Protein LYRIC	MTDH	63.80	85.78	8.59	4	4	4
O75607	Nucleoplasmin-3	NPM3	19.33	85.19	8.99	1	1	1
Q9NS69	itochondrial import receptor subunit TOM22 homo	TOMM22	15.51	85.03	16.20	2	2	2
P23246	Splicing factor, proline- and glutamine-rich	SFPQ	76.10	84.98	6.93	4	4	4
Q9BVI4	Nucleolar complex protein 4 homolog	NOC4L	58.43	84.92	9.30	3	3	3
P84090	Enhancer of rudimentary homolog	ERH	12.25	84.75	33.65	4	4	5
P24390	ER lumen protein-retaining receptor 1	KDELR1	24.53	84.33	5.19	1	1	2
A0A075B6S2	Immunoglobulin kappa variable 2D-29	IGKV2D-29	13.14	83.96	16.67	2	2	2
Q9Y5V3	Melanoma-associated antigen D1	MAGED1	86.11	83.70	1.93	1	1	1
Q15393	Splicing factor 3B subunit 3	SF3B3	135.49	83.05	4.77	5	5	5
Q7Z7K6	Centromere protein V	CENPV	29.93	82.02	9.45	2	2	2
P55060	Exportin-2	CSE1L	110.35	80.71	1.75	1	1	1
Q9Y520	Protein PRRC2C	PRRC2C	316.72	80.57	1.14	2	3	4
P62910	60S ribosomal protein L32	RPL32	15.85	80.30	28.15	4	4	4
P05141	ADP/ATP translocase 2	SLC25A5	32.83	80.08	13.09	4	4	4
Q9H307	Pinin	PNN	81.58	79.45	5.16	3	3	3
Q9H0D6	5'-3' exoribonuclease 2	XRN2	108.51	78.94	6.21	5	5	5
P0DP23	Calmodulin-1	CALM1	16.83	78.87	16.11	3	3	4
Q96P70	Importin-9	IPO9	115.89	77.59	2.59	2	2	2
Q92616	eIF-2-alpha kinase activator GCN1	GCN1	292.57	77.18	0.71	1	1	2
Q99848	Probable rRNA-processing protein EBP2	EBNA1BP2	34.83	77.12	7.19	1	1	1
Q58FF6	Putative heat shock protein HSP 90-beta 4	HSP90AB4P	58.23	75.86	8.32	1	4	4

Q92522	Histone H1.10	H1-10	22.47	75.85	12.68	2	2	2
Q9P0J0	dehydrogenase [ubiquinone] 1 alpha subcomplex su	NDUFA13	16.69	74.81	18.06	2	2	2
Q9BRL6	Serine/arginine-rich splicing factor 8	SRSF8	32.27	74.58	10.64	1	3	3
O95782	AP-2 complex subunit alpha-1	AP2A1	107.48	74.45	2.97	2	2	2
Q86YZ3	Hornerin	HRNR	282.23	74.29	3.58	2	2	2
O14925	ondrial import inner membrane translocase subunit	TIMM23	21.93	74.17	8.13	1	1	1
P02768	Albumin	ALB	69.32	74.01	2.46	1	1	2
P08559	rogenase E1 component subunit alpha, somatic forr	PDHA1	43.27	73.94	3.33	1	1	1
Q14694	Ubiquitin carboxyl-terminal hydrolase 10	USP10	87.08	73.85	3.38	2	2	2
Q14980	Nuclear mitotic apparatus protein 1	NUMA1	238.12	73.51	1.51	2	2	2
P46782	40S ribosomal protein S5	RPS5	22.86	73.33	25.00	5	5	6
Q99873	Protein arginine N-methyltransferase 1	PRMT1	42.43	72.69	3.23	1	1	1
O95232	Luc7-like protein 3	LUC7L3	51.44	72.39	6.94	2	2	2
P49458	Signal recognition particle 9 kDa protein	SRP9	10.11	72.27	22.09	2	2	2
P62820	Ras-related protein Rab-1A	RAB1A	22.66	72.13	13.17	2	2	2
Q9UJZ1	Stomatin-like protein 2, mitochondrial	STOML2	38.51	72.04	4.21	1	1	1
A4D1E9	GTP-binding protein 10	GTPBP10	42.91	71.80	6.20	2	2	2
P49756	RNA-binding protein 25	RBM25	100.12	71.75	9.25	6	6	6
Q86VM9	Zinc finger CCCH domain-containing protein 18	ZC3H18	106.32	71.64	1.99	1	1	1
Q5RKV6	Exosome complex component MTR3	EXOSC6	28.22	71.63	11.40	2	2	2
P41250	Glycine--tRNA ligase	GARS1	83.11	71.45	1.76	1	1	1
P13010	X-ray repair cross-complementing protein 5	XRCC5	82.65	70.95	4.64	2	2	2
P59998	Actin-related protein 2/3 complex subunit 4	ARPC4	19.65	70.57	21.43	4	4	4
O43795	Unconventional myosin-Ib	MYO1B	131.90	69.76	3.70	4	4	4
Q8TEX9	Importin-4	IPO4	118.64	69.54	1.02	1	1	1
P28288	ATP-binding cassette sub-family D member 3	ABCD3	75.43	69.47	3.95	2	2	2
P62854	40S ribosomal protein S26	RPS26	13.01	69.28	32.17	3	3	3

P84103	Serine/arginine-rich splicing factor 3	SRSF3	19.32	69.23	14.02	1	2	3
P49327	Fatty acid synthase	FASN	273.25	68.80	0.68	1	1	1
Q9BQ67	Glutamate-rich WD repeat-containing protein 1	GRWD1	49.39	68.65	3.59	1	1	2
Q9BYN8	28S ribosomal protein S26, mitochondrial	MRPS26	24.20	68.22	14.15	2	2	2
Q9BY77	Polymerase delta-interacting protein 3	POLDIP3	46.06	68.05	10.93	4	4	4
O95373	Importin-7	IPO7	119.44	68.04	2.50	2	2	2
P0DOX8	Immunoglobulin lambda-1 light chain	---	22.82	68.03	7.41	3	3	3
P55209	Nucleosome assembly protein 1-like 1	NAP1L1	45.35	67.14	9.97	3	3	3
Q6P1L8	39S ribosomal protein L14, mitochondrial	MRPL14	15.94	67.00	15.17	2	2	3
P62857	40S ribosomal protein S28	RPS28	7.84	65.76	30.43	2	2	2
O00541	Pescadillo homolog	PES1	67.96	65.66	4.93	3	3	3
Q15024	Exosome complex component RRP42	EXOSC7	31.80	65.14	5.15	1	1	1
Q9BQ39	ATP-dependent RNA helicase DDX50	DDX50	82.51	64.80	5.16	2	3	3
Q86U38	Nucleolar protein 9	NOP9	69.39	64.49	2.36	1	1	1
P19525	on-induced, double-stranded RNA-activated protei	EIF2AK2	62.06	63.78	4.72	2	2	2
P56134	ATP synthase subunit f, mitochondrial	ATP5MF	10.91	63.43	11.70	1	1	1
P42766	60S ribosomal protein L35	RPL35	14.54	63.26	22.76	4	4	6
P04004	Vitronectin	VTN	54.27	63.20	3.14	1	1	1
P08243	Asparagine synthetase [glutamine-hydrolyzing]	ASNS	64.33	62.79	2.50	1	1	1
P55072	Transitional endoplasmic reticulum ATPase	VCP	89.27	62.77	2.11	1	1	1
P62195	26S proteasome regulatory subunit 8	PSMC5	45.60	62.68	3.20	1	1	1
P24539	TP synthase F(0) complex subunit B1, mitochondri	ATP5PB	28.89	62.63	4.69	1	1	1
P38919	Eukaryotic initiation factor 4A-III	EIF4A3	46.84	62.37	8.03	3	3	4
Q9Y4Y9	U6 snRNA-associated Sm-like protein LSm5	LSM5	9.93	62.32	20.88	1	1	1
Q9NZM5	Ribosome biogenesis protein NOP53	NOP53	54.36	62.14	3.77	1	1	1
P42285	Exosome RNA helicase MTR4	MTREX	117.73	62.09	2.40	2	2	2
Q86WJ1	Chromodomain-helicase-DNA-binding protein 1-lik	CHD1L	100.94	61.91	3.23	2	2	2

P35251	Replication factor C subunit 1	RFC1	128.18	61.21	2.00	2	2	2
Q12906	Interleukin enhancer-binding factor 3	ILF3	95.28	60.97	6.15	5	5	5
Q00610	Clathrin heavy chain 1	CLTC	191.49	60.05	1.25	2	2	2
P52434	directed RNA polymerases I, II, and III subunit RP	POLR2H	17.13	60.01	8.67	1	1	1
P48643	T-complex protein 1 subunit epsilon	CCT5	59.63	59.76	4.07	2	2	2
P48047	ATP synthase subunit O, mitochondrial	ATP5PO	23.26	59.37	12.21	2	2	2
P10599	Thioredoxin	TXN	11.73	59.18	12.38	1	1	1
P23528	Cofilin-1	CFL1	18.49	59.13	18.67	2	2	2
Q07021	t component 1 Q subcomponent-binding protein, m	C1QBP	31.34	58.97	4.96	2	2	2
O75152	Zinc finger CCCH domain-containing protein 11A	ZC3H11A	89.08	58.70	2.72	1	1	1
P07195	L-lactate dehydrogenase B chain	LDHB	36.62	58.58	4.49	1	1	1
Q92552	28S ribosomal protein S27, mitochondrial	MRPS27	47.58	58.45	6.52	2	2	2
P60891	Ribose-phosphate pyrophosphokinase 1	PRPS1	34.81	58.44	12.89	4	4	4
O60832	H/ACA ribonucleoprotein complex subunit DKC1	DKC1	57.64	58.26	8.56	3	3	3
Q3ZCQ8	ondrial import inner membrane translocase subunit	TIMM50	39.62	57.40	7.93	2	2	2
Q9NZ01	Very-long-chain enoyl-CoA reductase	TECR	36.01	57.19	10.06	3	3	3
Q86YT6	E3 ubiquitin-protein ligase MIB1	MIB1	110.07	56.98	1.29	1	1	1
O95573	Long-chain-fatty-acid--CoA ligase 3	ACSL3	80.37	56.86	2.50	1	1	1
Q96T60	Bifunctional polynucleotide phosphatase/kinase	PNKP	57.04	56.59	4.41	2	2	2
P19623	Spermidine synthase	SRM	33.80	56.39	2.65	1	1	1
Q3MHD2	Protein LSM12 homolog	LSM12	21.69	56.35	7.18	1	1	1
Q9NYV4	Cyclin-dependent kinase 12	CDK12	164.05	55.90	1.28	2	2	2
P12004	Proliferating cell nuclear antigen	PCNA	28.75	55.89	16.09	4	4	4
P62318	Small nuclear ribonucleoprotein Sm D3	SNRPD3	13.91	55.76	15.08	2	2	2
P17987	T-complex protein 1 subunit alpha	TCP1	60.31	55.51	2.16	1	1	1
Q2M1P5	Kinesin-like protein KIF7	KIF7	150.50	55.38	1.34	1	1	1
Q9P2I0	avage and polyadenylation specificity factor subun	CPSF2	88.43	55.36	1.66	1	1	1

Q8N3R9	MAGUK p55 subfamily member 5	MPP5	77.25	55.30	1.78	1	1	1
Q9BRX2	Protein pelota homolog	PELO	43.33	55.02	3.64	1	1	1
O00410	Importin-5	IPO5	123.55	54.89	1.82	1	1	1
Q86VP6	Cullin-associated NEDD8-dissociated protein 1	CAND1	136.29	54.58	1.95	2	2	2
Q9H7B2	Ribosome production factor 2 homolog	RPF2	35.56	54.40	5.56	1	1	1
Q49A26	Putative oxidoreductase GLYR1	GLYR1	60.51	54.40	2.35	1	1	1
P61204	ADP-ribosylation factor 3	ARF3	20.59	54.33	4.42	1	1	1
Q8IZL8	Proline-, glutamic acid- and leucine-rich protein 1	PELP1	119.62	54.04	1.24	1	1	1
O15143	Actin-related protein 2/3 complex subunit 1B	ARPC1B	40.92	53.04	7.53	2	2	2
Q5JTH9	RRP12-like protein	RRP12	143.61	52.85	3.32	3	3	3
P60468	Protein transport protein Sec61 subunit beta	SEC61B	9.97	52.81	15.63	1	1	1
Q9UKD2	mRNA turnover protein 4 homolog	MRTO4	27.54	52.72	6.28	1	1	2
P46087	able 28S rRNA (cytosine(4447)-C(5))-methyltransf	NOP2	89.25	52.61	3.08	2	2	2
Q8NEJ9	Neuroguidin	NGDN	35.87	52.34	5.71	1	1	1
Q9H223	EH domain-containing protein 4	EHD4	61.14	52.29	2.03	1	1	1
P61927	60S ribosomal protein L37	RPL37	11.07	52.03	7.22	2	2	2
Q5SW79	Centrosomal protein of 170 kDa	CEP170	175.19	50.96	1.01	1	1	1
Q9H7E9	UPF0488 protein C8orf33	C8orf33	24.98	50.94	9.61	2	2	2
Q6P3X3	Tetratricopeptide repeat protein 27	TTC27	96.57	50.84	1.54	1	1	1
Q9C0J8	pre-mRNA 3' end processing protein WDR33	WDR33	145.80	50.74	0.97	1	1	1
Q9NVP1	ATP-dependent RNA helicase DDX18	DDX18	75.36	50.73	1.49	1	1	1
Q9GZR7	ATP-dependent RNA helicase DDX24	DDX24	96.27	50.62	1.63	1	1	1
Q9BSD7	Cancer-related nucleoside-triphosphatase	NTPCR	20.70	50.45	12.11	2	2	2
P51398	28S ribosomal protein S29, mitochondrial	DAP3	45.54	50.37	5.03	1	1	1
O15226	NF-kappa-B-repressing factor	NKRF	77.62	50.18	1.59	1	1	1
O95433	ivator of 90 kDa heat shock protein ATPase homol	AHSA1	38.25	50.13	6.51	1	1	1
O95299	genase [ubiquinone] 1 alpha subcomplex subunit 1	NDUFA10	40.73	49.91	6.20	1	1	1

Q96BW9	Phosphatidate cytidylyltransferase, mitochondrial	TAMM41	51.03	49.86	2.88	1	1	1
P50990	T-complex protein 1 subunit theta	CCT8	59.58	49.83	1.82	1	1	1
Q9H6R4	Nucleolar protein 6	NOL6	127.51	49.60	1.40	1	1	1
P53680	AP-2 complex subunit sigma	AP2S1	17.01	49.38	4.93	1	1	1
O43390	Heterogeneous nuclear ribonucleoprotein R	HNRNPR	70.90	49.35	4.74	2	2	2
P18085	ADP-ribosylation factor 4	ARF4	20.50	49.19	4.44	1	1	1
Q15366	Poly(rC)-binding protein 2	PCBP2	38.56	49.15	6.58	2	2	2
Q8TB52	F-box only protein 30	FBXO30	82.25	48.54	1.88	1	1	1
A8MWD9	ative small nuclear ribonucleoprotein G-like protei	SNRPGP15	8.54	48.14	15.79	1	1	1
Q9Y295	Developmentally-regulated GTP-binding protein 1	DRG1	40.52	48.14	3.81	1	1	1
O15381	Nuclear valosin-containing protein-like	NVL	94.99	47.64	2.80	2	2	2
Q12788	Transducin beta-like protein 3	TBL3	88.98	47.58	4.21	3	3	3
P82933	28S ribosomal protein S9, mitochondrial	MRPS9	45.81	47.37	3.28	1	1	1
Q6P158	Putative ATP-dependent RNA helicase DHX57	DHX57	155.51	46.66	1.01	1	1	1
Q9NXF1	Testis-expressed protein 10	TEX10	105.61	46.57	2.15	2	2	2
P38646	Stress-70 protein, mitochondrial	HSPA9	73.64	46.45	3.98	2	2	2
Q14103	Heterogeneous nuclear ribonucleoprotein D0	HNRNPD	38.41	46.43	3.94	1	1	1
Q9BVJ6	small nucleolar RNA-associated protein 14 homolo	UTP14A	87.92	46.09	1.43	1	1	1
Q86W42	THO complex subunit 6 homolog	THOC6	37.51	46.08	5.57	1	1	1
Q9NY93	Probable ATP-dependent RNA helicase DDX56	DDX56	61.55	46.07	5.85	3	3	3
Q8NB90	ATPase family protein 2 homolog	SPATA5	97.84	46.05	1.34	1	1	1
Q01780	Exosome component 10	EXOSC10	100.77	45.91	1.47	1	1	1
P62826	GTP-binding nuclear protein Ran	RAN	24.41	45.79	13.89	3	3	3
P07741	Adenine phosphoribosyltransferase	APRT	19.60	45.64	7.22	1	1	1
P52907	F-actin-capping protein subunit alpha-1	CAPZA1	32.90	45.50	8.74	2	2	2
P53618	Coatomer subunit beta	COPB1	107.07	45.47	2.52	2	2	2
P16989	Y-box-binding protein 3	YBX3	40.07	45.43	9.95	1	2	2

Q9NQT5	Exosome complex component RRP40	EXOSC3	29.55	45.34	5.45	1	1	1
Q9H2U1	ATP-dependent DNA/RNA helicase DHX36	DHX36	114.69	45.30	1.39	1	1	1
Q9Y2X9	Zinc finger protein 281	ZNF281	96.86	45.29	3.80	2	2	2
P08574	Cytochrome c1, heme protein, mitochondrial	CYC1	35.40	45.24	2.15	1	1	1
P32119	Peroxiredoxin-2	PRDX2	21.88	45.14	8.59	1	1	1
O94906	Pre-mRNA-processing factor 6	PRPF6	106.86	45.13	0.96	1	1	1
P83881	60S ribosomal protein L36a	RPL36A	12.43	44.91	7.55	1	1	1
Q8NI27	THO complex subunit 2	THOC2	182.66	44.91	1.07	2	2	2
P07814	Bifunctional glutamate/proline--tRNA ligase	EPRS1	170.48	44.88	1.52	2	2	2
O14980	Exportin-1	XPO1	123.31	44.42	3.45	3	3	3
Q7KZI7	Serine/threonine-protein kinase MARK2	MARK2	87.86	44.39	2.54	1	1	1
P81605	Dermcidin	DCD	11.28	43.96	20.00	2	2	2
P62899	60S ribosomal protein L31	RPL31	14.45	43.86	17.60	2	2	2
Q14241	Elongin-A	ELOA	89.85	43.82	2.01	1	1	1
P18887	DNA repair protein XRCC1	XRCC1	69.43	43.78	1.58	1	1	1
P41252	Isoleucine--tRNA ligase, cytoplasmic	IARS1	144.41	43.61	1.66	2	2	2
Q9Y2Z2	Protein MTO1 homolog, mitochondrial	MTO1	79.91	43.49	1.95	1	1	1
O95831	Apoptosis-inducing factor 1, mitochondrial	AIFM1	66.86	43.36	2.45	1	1	1
Q9UBL6	Copine-7	CPNE7	70.25	43.28	1.42	1	1	1
O00264	mbrane-associated progesterone receptor componer	PGRMC1	21.66	43.26	4.10	1	1	1
O43592	Exportin-T	XPOT	109.89	43.04	2.49	2	2	2
Q12996	Cleavage stimulation factor subunit 3	CSTF3	82.87	42.68	2.65	1	1	1
Q9BZF1	Oxysterol-binding protein-related protein 8	OSBPL8	101.13	42.51	1.24	1	1	1
Q9BXS6	Nucleolar and spindle-associated protein 1	NUSAP1	49.42	42.45	2.72	1	1	1
Q9BWJ5	Splicing factor 3B subunit 5	SF3B5	10.13	42.41	12.79	1	1	1
Q3KQU3	MAP7 domain-containing protein 1	MAP7D1	92.76	42.23	1.90	1	1	1
Q14978	Nucleolar and coiled-body phosphoprotein 1	NOLC1	73.56	42.02	3.29	2	2	2

P49207	60S ribosomal protein L34	RPL34	13.28	41.99	15.38	2	2	2
Q04637	Eukaryotic translation initiation factor 4 gamma 1	EIF4G1	175.38	41.93	0.69	1	1	1
Q8NI36	WD repeat-containing protein 36	WDR36	105.26	41.92	3.68	3	3	3
Q15154	Pericentriolar material 1 protein	PCM1	228.40	41.85	0.54	1	1	1
P62891	60S ribosomal protein L39	RPL39	6.40	41.68	19.61	2	2	2
P18077	60S ribosomal protein L35a	RPL35A	12.53	41.65	25.45	3	3	3
Q06265	Exosome complex component RRP45	EXOSC9	48.92	41.46	2.28	1	1	1
Q9UM00	Calcium load-activated calcium channel	TMCO1	27.06	41.35	6.28	1	1	1
O60783	28S ribosomal protein S14, mitochondrial	MRPS14	15.13	41.15	10.16	1	1	1
Q13523	Serine/threonine-protein kinase PRP4 homolog	PRPF4B	116.92	40.63	1.19	1	1	1
Q8TDD1	ATP-dependent RNA helicase DDX54	DDX54	98.53	40.44	3.75	3	3	3
Q7Z478	ATP-dependent RNA helicase DHX29	DHX29	155.14	40.33	0.66	1	1	1
Q6PEV8	Protein FAM199X	FAM199X	42.78	40.32	3.87	1	1	1
P55265	Double-stranded RNA-specific adenosine deaminase	ADAR	135.98	40.30	1.96	2	2	2
P0C0S5	Histone H2A.Z	H2AZ1	13.55	40.23	14.84	1	2	3
Q9UNZ5	Leydig cell tumor 10 kDa protein homolog	C19orf53	10.57	40.03	9.09	1	1	1
Q12965	Unconventional myosin-Ie	MYO1E	126.98	39.85	1.62	2	2	2
Q7Z417	clear fragile X mental retardation-interacting protei	NUFIP2	76.08	39.79	4.75	2	2	2
Q9Y5B6	PAX3- and PAX7-binding protein 1	PAXBP1	104.74	39.78	1.31	1	1	1
P38432	Coilin	COIL	62.57	39.72	2.95	1	1	1
Q5C9Z4	Nucleolar MIF4G domain-containing protein 1	NOM1	96.20	39.50	1.40	1	1	1
Q6UN15	Pre-mRNA 3'-end-processing factor FIP1	FIP1L1	66.49	39.14	3.20	1	1	1
Q13501	Sequestosome-1	SQSTM1	47.66	39.13	3.18	1	1	1
Q8N612	FTS and Hook-interacting protein	FAM160A2	105.50	39.08	0.62	1	1	1
O95816	BAG family molecular chaperone regulator 2	BAG2	23.76	38.92	4.27	1	1	1
Q92747	Actin-related protein 2/3 complex subunit 1A	ARPC1A	41.54	38.87	5.95	2	2	2
Q15758	Neutral amino acid transporter B(0)	SLC1A5	56.56	38.73	3.70	1	1	1

Q9UMY1	Nucleolar protein 7	NOL7	29.41	38.73	3.89	1	1	1
Q12894	Interferon-related developmental regulator 2	IFRD2	54.78	38.19	1.78	1	1	1
P00403	Cytochrome c oxidase subunit 2	MT-CO2	25.55	38.00	13.22	2	2	2
Q9BV38	WD repeat-containing protein 18	WDR18	47.38	37.84	4.17	2	2	2
Q14692	Ribosome biogenesis protein BMS1 homolog	BMS1	145.72	37.76	1.25	1	1	1
Q8N5H7	SH2 domain-containing protein 3C	SH2D3C	94.35	37.68	0.70	1	1	1
Q9NRL2	omodomain adjacent to zinc finger domain protein	BAZ1A	178.59	37.66	0.71	1	1	1
Q9H1I8	Activating signal cointegrator 1 complex subunit 2	ASCC2	86.31	37.54	1.72	1	1	1
Q86XZ4	Spermatogenesis-associated serine-rich protein 2	SPATS2	59.51	37.19	2.75	1	1	1
P57678	Gem-associated protein 4	GEMIN4	119.96	37.07	1.42	1	1	1
O75964	ATP synthase subunit g, mitochondrial	ATP5MG	11.42	36.75	23.30	2	2	2
Q12789	General transcription factor 3C polypeptide 1	GTF3C1	238.73	36.68	0.76	1	1	1
P55884	Eukaryotic translation initiation factor 3 subunit B	EIF3B	92.42	36.63	1.72	1	1	1
Q8WTT2	Nucleolar complex protein 3 homolog	NOC3L	92.49	36.51	1.63	1	1	1
Q9BSJ2	Gamma-tubulin complex component 2	TUBGCP2	102.47	36.46	1.22	1	1	1
Q9P031	Thyroid transcription factor 1-associated protein 26	CCDC59	28.65	36.35	4.15	1	1	1
Q16718	dehydrogenase [ubiquinone] 1 alpha subcomplex su	NDUFA5	13.45	36.26	8.62	1	1	1
P54136	Arginine--tRNA ligase, cytoplasmic	RARS1	75.33	36.02	1.52	1	1	1
Q9BUJ2	terogeneous nuclear ribonucleoprotein U-like protei	HNRNPUL1	95.68	35.95	0.82	1	1	1
O00767	Acyl-CoA desaturase	SCD	41.50	35.80	3.34	1	1	2
P46013	Proliferation marker protein Ki-67	MKI67	358.47	35.71	0.61	1	1	1
Q5BJF2	Sigma intracellular receptor 2	TMEM97	20.83	35.63	4.55	1	1	1
O15235	28S ribosomal protein S12, mitochondrial	MRPS12	15.16	35.59	7.97	1	1	1
P14678	l nuclear ribonucleoprotein-associated proteins B a	SNRPB	24.59	35.47	2.92	1	1	1
P68431	Histone H3.1	H3C1	15.39	35.42	16.91	1	3	3
Q16637	Survival motor neuron protein	SMN1	31.83	35.11	3.40	1	1	1
P45973	Chromobox protein homolog 5	CBX5	22.21	34.93	5.76	1	1	1

Q9Y4W2	Ribosomal biogenesis protein LAS1L	LAS1L	83.01	34.88	1.91	1	1	1
O60784	Target of Myb protein 1	TOM1	53.79	34.86	5.28	2	2	2
O15371	Eukaryotic translation initiation factor 3 subunit D	EIF3D	63.93	34.65	2.55	1	1	1
Q9NWB6	Arginine and glutamate-rich protein 1	ARGLU1	33.20	34.64	5.86	2	2	2
Q5XKP0	MICOS complex subunit MIC13	MICOS13	13.08	34.49	5.08	1	1	1
Q15365	Poly(rC)-binding protein 1	PCBP1	37.47	34.37	3.65	1	1	1
O75400	Pre-mRNA-processing factor 40 homolog A	PRPF40A	108.74	34.20	1.78	1	1	1
Q13868	Exosome complex component RRP4	EXOSC2	32.77	34.00	5.12	1	1	1
Q96IZ0	PRKC apoptosis WT1 regulator protein	PAWR	36.55	33.35	3.82	1	1	1
Q7Z4Q2	HEAT repeat-containing protein 3	HEATR3	74.54	33.28	2.35	1	1	1
E9PRG8	Uncharacterized protein C11orf98	C11orf98	14.23	33.24	26.02	2	3	3
Q14126	Desmoglein-2	DSG2	122.22	33.22	1.25	1	1	1
Q9H840	Gem-associated protein 7	GEMIN7	14.53	33.15	9.92	1	1	1
O14979	Heterogeneous nuclear ribonucleoprotein D-like	HNRNPDL	46.41	33.11	3.81	1	1	1
Q9BRJ7	Tudor-interacting repair regulator protein	NUDT16L1	23.32	32.88	6.64	1	1	1
P02794	Ferritin heavy chain	FTH1	21.21	32.78	8.20	1	1	1
P61962	DDB1- and CUL4-associated factor 7	DCAF7	38.90	32.73	4.39	1	1	1
Q9Y3D9	28S ribosomal protein S23, mitochondrial	MRPS23	21.76	32.63	5.26	1	1	1
O15020	Spectrin beta chain, non-erythrocytic 2	SPTBN2	271.16	32.58	0.25	1	1	1
Q01484	Ankyrin-2	ANK2	433.45	32.56	0.20	1	1	1
P57088	Transmembrane protein 33	TMEM33	27.96	32.51	3.64	1	1	1
Q92769	Histone deacetylase 2	HDAC2	55.33	32.39	2.46	1	1	1
P12956	X-ray repair cross-complementing protein 6	XRCC6	69.80	32.37	3.28	1	1	1
Q9NUL7	Probable ATP-dependent RNA helicase DDX28	DDX28	59.55	32.35	2.04	1	1	1
P40937	Replication factor C subunit 5	RFC5	38.47	32.21	2.94	1	1	1
Q6WCQ1	Myosin phosphatase Rho-interacting protein	MPRIP	116.46	32.10	2.24	2	2	2
Q9Y3C1	Nucleolar protein 16	NOP16	21.18	32.05	6.18	1	1	1

Q9UBB4	Ataxin-10	ATXN10	53.46	31.98	5.26	2	2	2
Q9H6S0	3'-5' RNA helicase YTHDC2	YTHDC2	160.15	31.89	1.40	2	2	2
Q9C0D0	Phosphatase and actin regulator 1	PHACTR1	66.27	31.81	1.55	1	1	1
P40939	Trifunctional enzyme subunit alpha, mitochondrial	HADHA	82.95	31.74	2.75	1	1	1
O95793	ıble-stranded RNA-binding protein Staufen homolc	STAU1	63.14	31.62	1.73	1	1	1
Q9Y3D8	Adenylate kinase isoenzyme 6	AK6	20.05	31.50	5.23	1	1	1
Q5TEC6	Histone HIST2H3PS2	H3-2	15.42	31.43	19.85	1	3	3
P82650	28S ribosomal protein S22, mitochondrial	MRPS22	41.25	31.38	2.22	1	1	1
O60716	Catenin delta-1	CTNND1	108.10	31.18	1.34	1	1	1
Q9Y3B4	Splicing factor 3B subunit 6	SF3B6	14.58	31.15	11.20	1	1	1
Q04837	single-stranded DNA-binding protein, mitochondria	SSBP1	17.25	31.08	10.14	1	1	1
O43175	D-3-phosphoglycerate dehydrogenase	PHGDH	56.61	31.06	1.69	1	1	1
Q9ULH7	Myocardin-related transcription factor B	MRTFB	118.05	30.66	0.55	1	1	1
P40938	Replication factor C subunit 3	RFC3	40.53	30.65	3.09	1	1	1
Q14315	Filamin-C	FLNC	290.84	30.33	0.40	1	1	1
Q9BW72	HIG1 domain family member 2A, mitochondrial	HIGD2A	11.52	30.28	27.36	1	1	1
P52294	Importin subunit alpha-5	KPNA1	60.18	30.23	3.35	1	1	1
P62714	eonine-protein phosphatase 2A catalytic subunit be	PPP2CB	35.55	29.92	3.56	1	1	1
P51151	Ras-related protein Rab-9A	RAB9A	22.82	29.65	6.47	1	1	1
Q15651	›ility group nucleosome-binding domain-containing	HMGN3	10.66	29.61	9.09	1	1	1
Q05519	Serine/arginine-rich splicing factor 11	SRSF11	53.51	29.40	4.55	2	2	2
P63220	40S ribosomal protein S21	RPS21	9.11	29.39	18.07	1	1	1
P82930	28S ribosomal protein S34, mitochondrial	MRPS34	25.63	29.31	3.67	1	1	1
Q8WVM0	Dimethyladenosine transferase 1, mitochondrial	TFB1M	39.52	29.10	2.31	1	1	1
Q9UBU9	Nuclear RNA export factor 1	NXF1	70.14	28.82	1.94	1	1	1
Q14137	Ribosome biogenesis protein BOP1	BOP1	83.58	28.65	3.35	1	1	1
Q12874	Splicing factor 3A subunit 3	SF3A3	58.81	28.65	1.80	1	1	1

Q8WUK0	tidylglycerophosphatase and protein-tyrosine phosp	PTPMT1	22.83	28.30	5.97	1	1	1
Q5VTL8	Pre-mRNA-splicing factor 38B	PRPF38B	64.43	28.29	1.83	1	1	1
P06576	ATP synthase subunit beta, mitochondrial	ATP5F1B	56.53	28.10	2.46	1	1	1
Q5SSJ5	Heterochromatin protein 1-binding protein 3	HP1BP3	61.17	27.76	1.45	1	1	1
O00743	rine/threonine-protein phosphatase 6 catalytic subu	PPP6C	35.12	27.69	2.95	1	1	1
Q9BT67	NEDD4 family-interacting protein 1	NDFIP1	24.88	27.38	7.69	1	1	1
Q16795	ɔgenase [ubiquinone] 1 alpha subcomplex subunit 9	NDUFA9	42.48	27.37	4.51	1	1	1
Q9BZL1	Ubiquitin-like protein 5	UBL5	8.54	27.28	12.33	1	1	1
P62304	Small nuclear ribonucleoprotein E	SNRPE	10.80	26.64	11.96	1	1	1
Q9NTJ3	Structural maintenance of chromosomes protein 4	SMC4	147.09	26.46	0.78	1	1	1
Q9Y3U8	60S ribosomal protein L36	RPL36	12.25	26.41	8.57	1	1	1
P14618	Pyruvate kinase PKM	PKM	57.90	26.41	1.69	1	1	1
Q9UI10	Translation initiation factor eIF-2B subunit delta	EIF2B4	57.52	26.25	4.97	1	1	1
Q00577	Transcriptional activator protein Pur-alpha	PURA	34.89	25.81	2.80	1	1	1
P15924	Desmoplakin	DSP	331.57	25.78	0.49	1	1	1
Q9Y678	Coatomer subunit gamma-1	COPG1	97.66	25.54	1.37	1	1	1
O60231	˂NA-splicing factor ATP-dependent RNA helicase I	DHX16	119.19	25.48	1.15	1	1	1
Q9NW13	RNA-binding protein 28	RBM28	85.69	25.38	3.56	3	3	3
P09661	U2 small nuclear ribonucleoprotein A'	SNRPA1	28.40	25.16	3.14	1	1	1
O60942	mRNA-capping enzyme	RNGTT	68.51	24.97	2.01	1	1	1
Q96EY1	DnaJ homolog subfamily A member 3, mitochondria	DNAJA3	52.46	24.80	2.50	1	1	1
Q13148	TAR DNA-binding protein 43	TARDBP	44.71	24.72	2.90	1	1	1
O76031	lent Clp protease ATP-binding subunit clpX-like, m	CLPX	69.18	24.70	1.58	1	1	1
P55769	NHP2-like protein 1	SNU13	14.17	24.62	14.84	2	2	2
P39748	Flap endonuclease 1	FEN1	42.57	24.21	2.89	1	1	1
Q9Y6K0	Choline/ethanolaminephosphotransferase 1	CEPT1	46.52	24.06	2.64	1	1	1
Q14232	Translation initiation factor eIF-2B subunit alpha	EIF2B1	33.69	24.04	2.30	1	1	1

Q15014	Mortality factor 4-like protein 2	MORF4L2	32.29	24.02	3.82	1	1	1
P17302	Gap junction alpha-1 protein	GJA1	42.98	23.97	2.09	1	1	1
Q8N1F7	Nuclear pore complex protein Nup93	NUP93	93.43	23.79	1.34	1	1	1
P78345	Ribonuclease P protein subunit p38	RPP38	31.82	23.64	4.24	1	1	1
Q8WXX5	DnaJ homolog subfamily C member 9	DNAJC9	29.89	23.55	3.46	1	1	1
P63244	Receptor of activated protein C kinase 1	RACK1	35.06	23.50	6.31	2	2	2
P04181	Ornithine aminotransferase, mitochondrial	OAT	48.50	23.33	2.05	1	1	1
Q9H329	Band 4.1-like protein 4B	EPB41L4B	99.65	22.76	0.89	1	1	1
Q9Y277	Voltage-dependent anion-selective channel protein 3	VDAC3	30.64	22.54	4.59	1	1	1
Q9BRP1	Programmed cell death protein 2-like	PDCD2L	39.39	22.51	3.35	1	1	1
Q9BPX5	ctin-related protein 2/3 complex subunit 5-like prote	ARPC5L	16.93	22.45	3.92	1	1	1
O00487	26S proteasome non-ATPase regulatory subunit 14	PSMD14	34.56	22.34	2.26	1	1	1
Q96AG4	Leucine-rich repeat-containing protein 59	LRRC59	34.91	22.06	3.91	1	1	1
P78362	SRSF protein kinase 2	SRPK2	77.48	22.02	1.02	1	1	1
P20700	Lamin-B1	LMNB1	66.37	21.65	1.71	1	1	1
Q16531	DNA damage-binding protein 1	DDB1	126.89	21.30	0.70	1	1	1
O00488	Zinc finger protein 593	ZNF593	15.19	21.28	6.72	1	1	1
O60678	Protein arginine N-methyltransferase 3	PRMT3	59.87	20.93	2.64	1	1	1
Q969X5	asmic reticulum-Golgi intermediate compartment pr	ERGIC1	32.57	20.42	2.76	1	1	1
P06746	DNA polymerase beta	POLB	38.15	20.19	2.69	1	1	1
Q8IY81	re-rRNA 2'-O-ribose RNA methyltransferase FTSJ	FTSJ3	96.50	20.10	2.01	1	1	1
Q9UNX4	WD repeat-containing protein 3	WDR3	106.03	19.81	1.17	1	1	1
P01859	Immunoglobulin heavy constant gamma 2	IGHG2	35.88	19.51	2.76	1	1	1
P60842	Eukaryotic initiation factor 4A-I	EIF4A1	46.13	0.00	5.91	2	2	2
Q96T76	MMS19 nucleotide excision repair protein homolog	MMS19	113.22	0.00	2.23	1	1	1
Q96BN2	Transcriptional adapter 1	TADA1	37.36	0.00	3.28	1	1	1
Q9H845	Complex I assembly factor ACAD9, mitochondrial	ACAD9	68.72	0.00	2.42	1	1	1

Q13144	Translation initiation factor eIF-2B subunit epsilon	EIF2B5	80.33	0.00	1.66	1	1	1
Q53GQ0	Very-long-chain 3-oxoacyl-CoA reductase	HSD17B12	34.30	0.00	9.29	1	1	1
Q9UBI6	ucleotide-binding protein G(I)/G(S)/G(O) subunit g	GNG12	8.00	0.00	15.28	1	1	1
O15145	Actin-related protein 2/3 complex subunit 3	ARPC3	20.53	0.00	6.18	1	1	1
Q8NI77	Kinesin-like protein KIF18A	KIF18A	102.22	0.00	1.78	1	1	1
Q96DI7	U5 small nuclear ribonucleoprotein 40 kDa protein	SNRNP40	39.29	0.00	3.08	1	1	1
Q9UBB9	Tuftelin-interacting protein 11	TFIP11	96.76	0.00	1.79	1	1	1
P78316	Nucleolar protein 14	NOP14	97.61	0.00	1.05	1	1	1
Q15058	Kinesin-like protein KIF14	KIF14	186.38	0.00	0.91	1	1	1
Q92900	Regulator of nonsense transcripts 1	UPF1	124.27	0.00	0.97	1	1	1
O75165	DnaJ homolog subfamily C member 13	DNAJC13	254.25	0.00	0.53	1	1	1
Q7Z4L9	Protein phosphatase 1 regulatory subunit 42	PPP1R42	35.46	0.00	2.59	1	1	1
Q8WXA3	RUN and FYVE domain-containing protein 2	RUFY2	69.97	0.00	1.32	1	1	1
Q9HAU5	Regulator of nonsense transcripts 2	UPF2	147.72	0.00	0.55	1	1	1
Q7RTR2	NLR family CARD domain-containing protein 3	NLRC3	114.59	0.00	0.66	1	1	1
