# Supplementary material for: VHL suppresses UBE3B-mediated breast tumor growth and metastasis
Source: Cell Death Dis. 2024 Jun 24;15(6):446. doi: 10.1038/s41419-024-06844-x (PMC11196597; doi:10.1038/s41419-024-06844-x)

Fig.1 Raw data

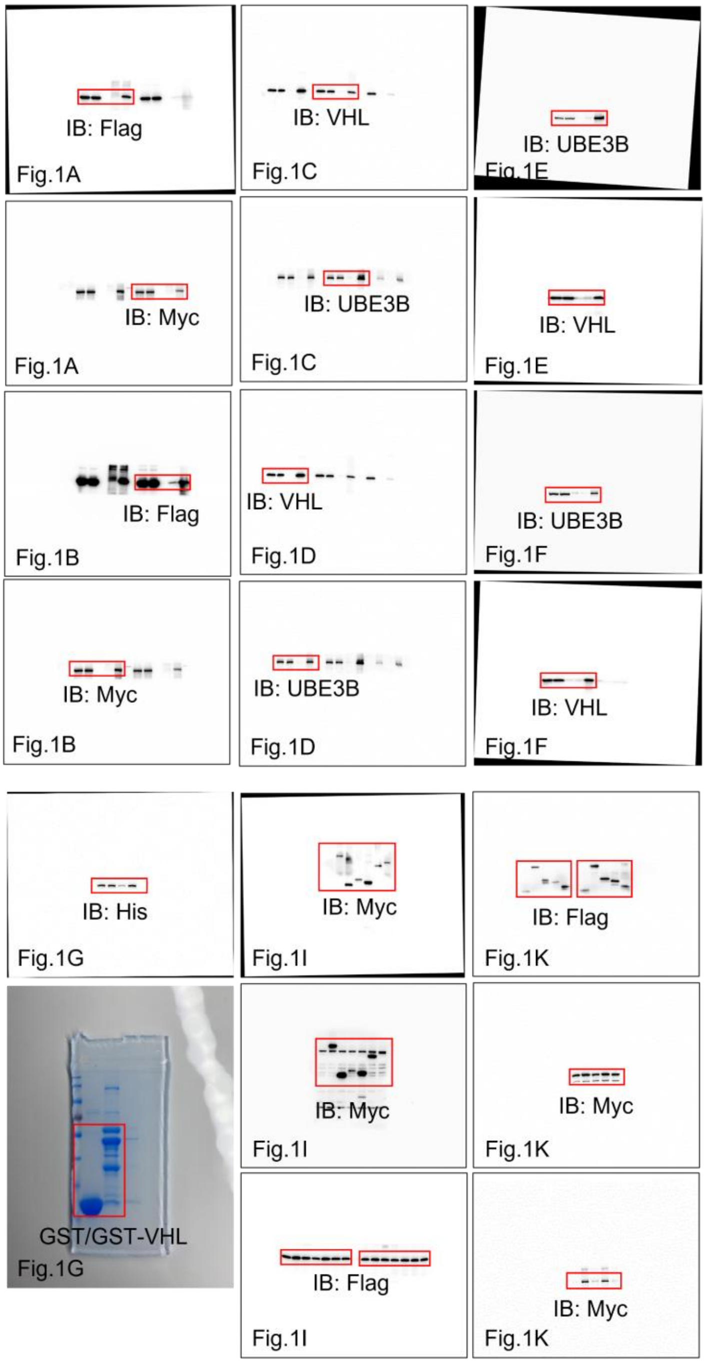

Fig.S1 Raw data

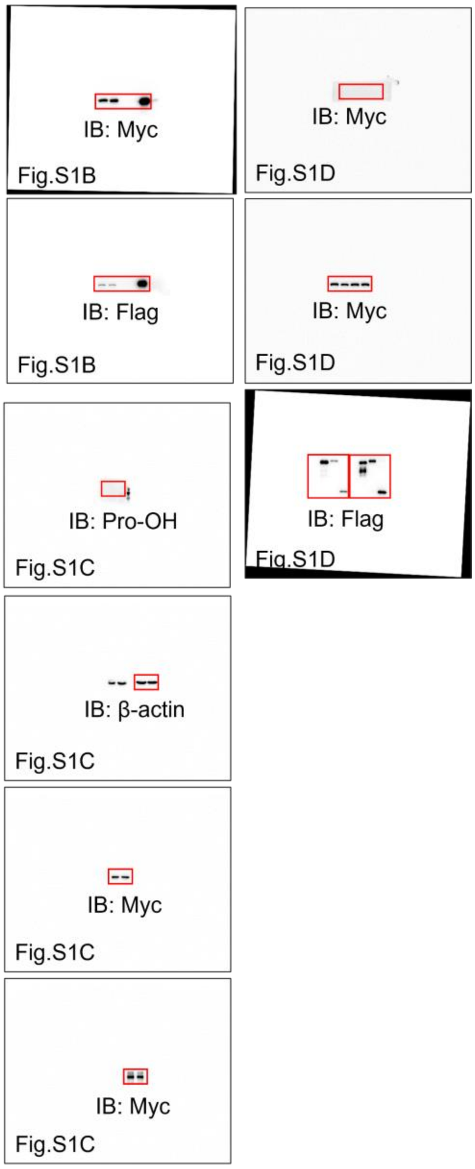

Fig.2 Raw data

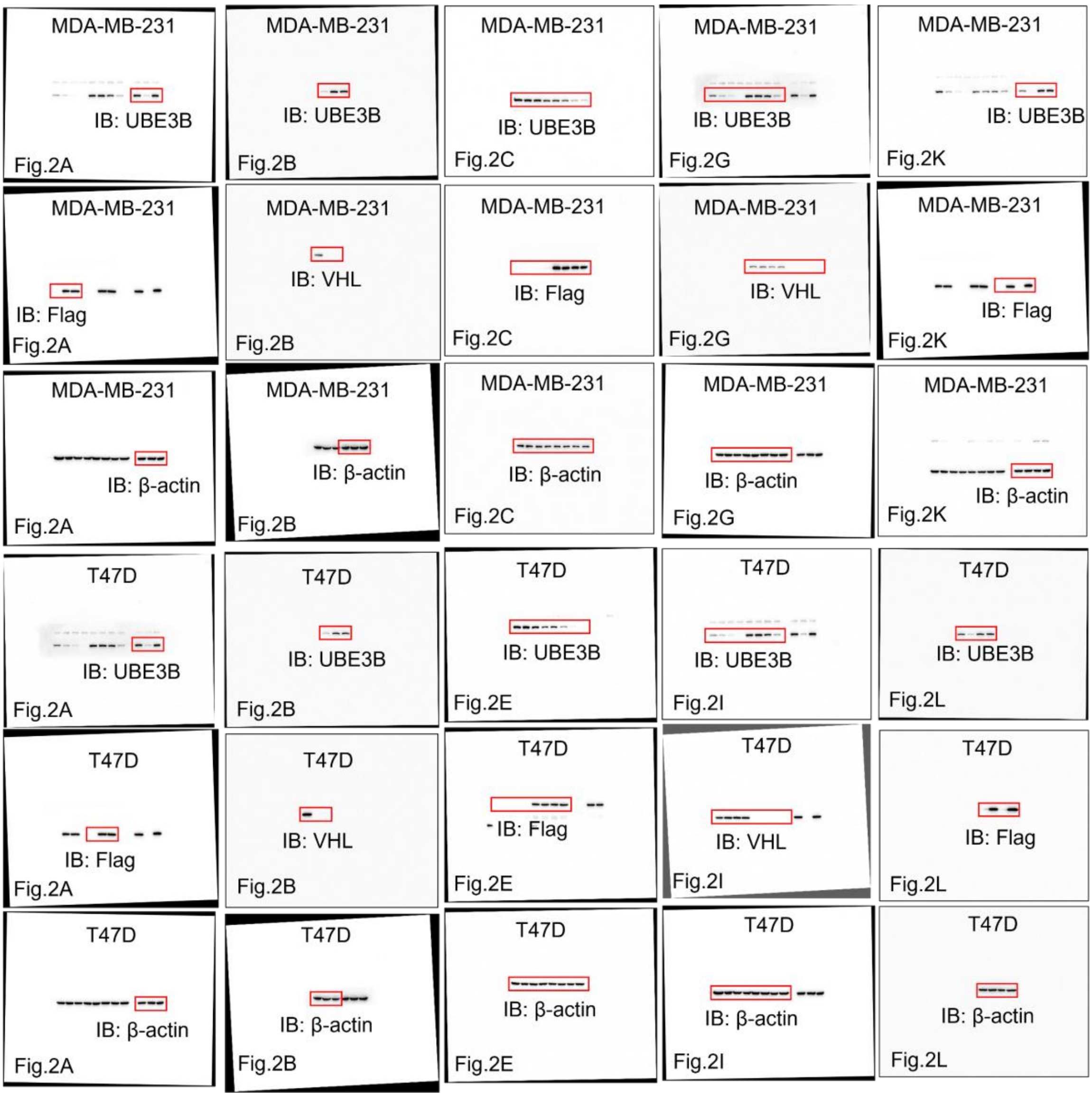

Fig.S2 Raw data

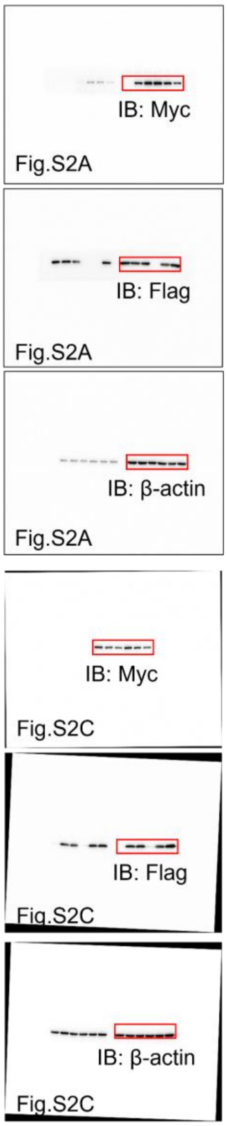

Fig.3 Raw data

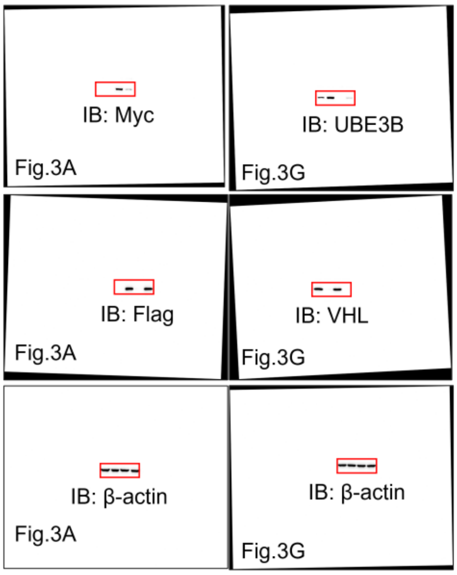

Fig.S3 Raw data

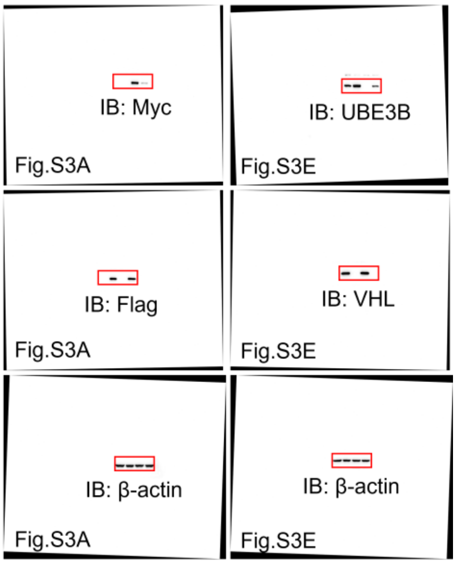

Fig.4 Raw data

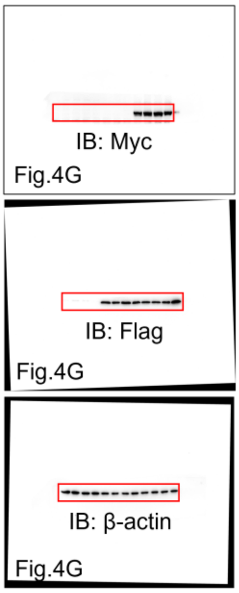

Fig.S4 Raw data

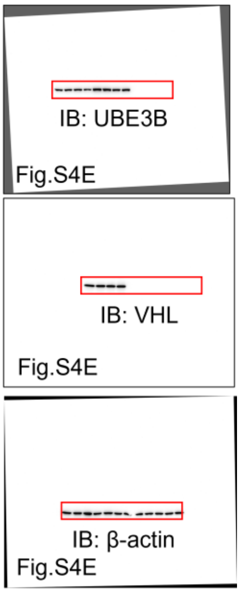

Fig.5 Raw data

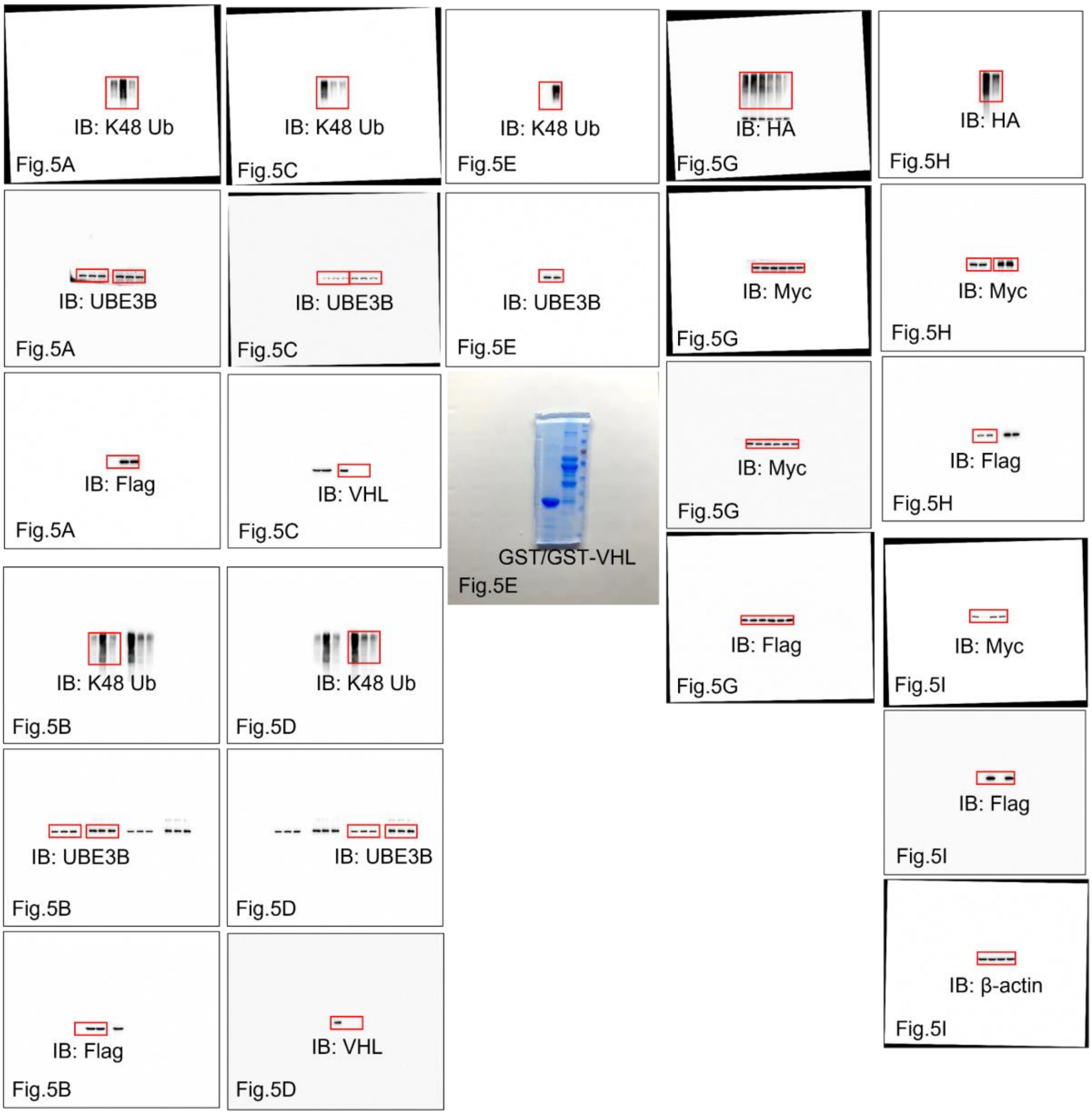

Fig.S5 Raw data

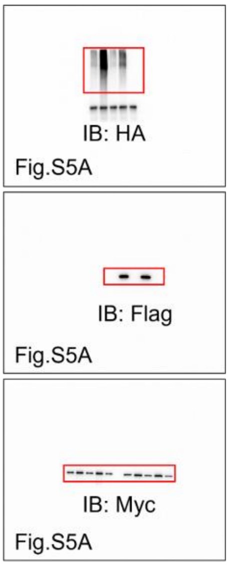

Fig.S6 Raw data

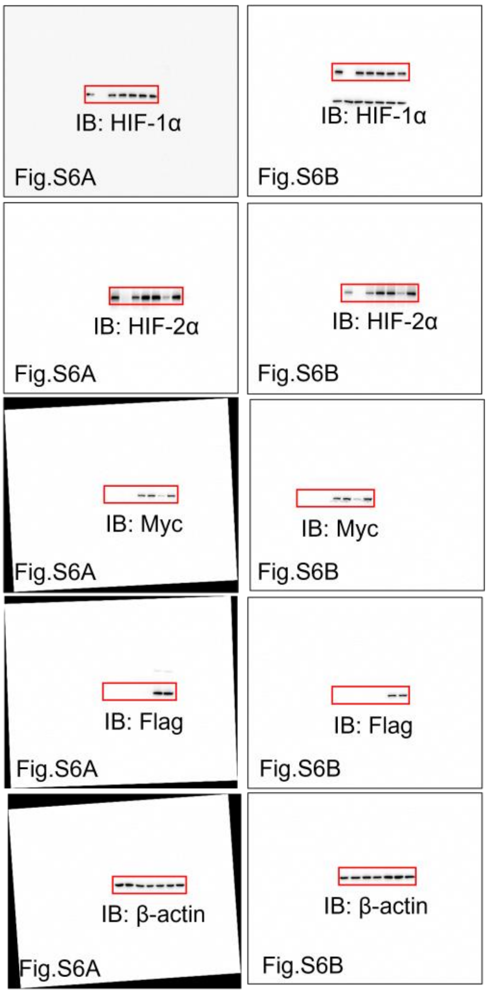

Fig.7 Raw data

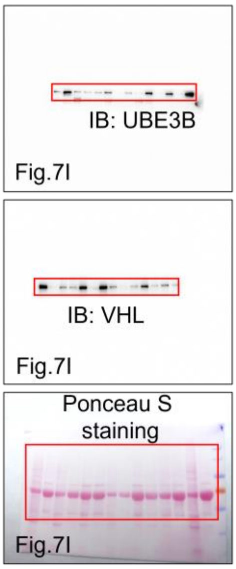

Fig.S7 Raw data

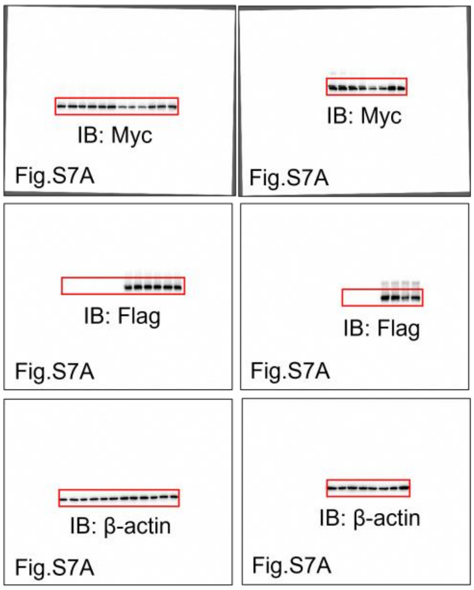

Supplement: Supplementary file 2 — Original Data [file 41419_2024_6844_MOESM2_ESM.pdf]
